# Mechanisms controlling membrane recruitment and activation of the autoinhibited SHIP1 inositol 5-phosphatase

**DOI:** 10.1016/j.jbc.2023.105022

**Published:** 2023-07-07

**Authors:** Grace L. Waddell, Emma E. Drew, Henry P. Rupp, Scott D. Hansen

**Affiliations:** 1 Department of Chemistry and Biochemistry, University of Oregon, Eugene, Oregon, USA; 2 Institute of Molecular Biology, University of Oregon, Eugene, Oregon, USA

**Keywords:** phosphatase, phosphatidylinositol-3-kinase, phosphatidylinositol signaling, phosphatidylinositol, phosphatidylinositol phosphatase, Src homology 2 domain, SHIP1

## Abstract

Signal transduction downstream of growth factor and immune receptor activation relies on the production of phosphatidylinositol-(3,4,5)-trisphosphate (PI(3,4,5)P_3_) lipids by PI3K. Regulating the strength and duration of PI3K signaling in immune cells, Src homology 2 domain–containing inositol 5-phosphatase 1 (SHIP1) controls the dephosphorylation of PI(3,4,5)P_3_ to generate phosphatidylinositol-(3,4)-bisphosphate. Although SHIP1 has been shown to regulate neutrophil chemotaxis, B-cell signaling, and cortical oscillations in mast cells, the role that lipid and protein interactions serve in controlling SHIP1 membrane recruitment and activity remains unclear. Using single-molecule total internal reflection fluorescence microscopy, we directly visualized membrane recruitment and activation of SHIP1 on supported lipid bilayers and the cellular plasma membrane. We find that localization of the central catalytic domain of SHIP1 is insensitive to dynamic changes in PI(3,4,5)P_3_ and phosphatidylinositol-(3,4)-bisphosphate both *in vitro* and *in vivo*. Very transient SHIP1 membrane interactions were detected only when membranes contained a combination of phosphatidylserine and PI(3,4,5)P_3_ lipids. Molecular dissection reveals that SHIP1 is autoinhibited with the N-terminal Src homology 2 domain playing a critical role in suppressing phosphatase activity. Robust SHIP1 membrane localization and relief of autoinhibition can be achieved through interactions with immunoreceptor-derived phosphopeptides presented either in solution or conjugated to a membrane. Overall, this work provides new mechanistic details concerning the dynamic interplay between lipid-binding specificity, protein–protein interactions, and the activation of autoinhibited SHIP1.

Phosphatidylinositol phosphate (PIP) lipids play a crucial function in eukaryotic cell biology by regulating the localization and activity of numerous signaling proteins on intracellular membranes (1). PIP lipids are transiently created by different classes of lipid kinases and phosphatases, which are activated during various cell signaling pathways (2). Understanding how PIP lipid–modifying enzymes are regulated is critical for determining how cells control the strength and duration of PIP lipid signaling in cell biology. Misregulation of PIP lipid homeostasis has been shown to profoundly affect cell growth and proliferation, which is linked to poor prognosis in numerous human diseases (3).

The Src homology 2 (SH2) domain–containing inositol 5-phosphatase 1 (SHIP1) is a hematopoietic cell–specific lipid phosphatase that dephosphorylates phosphatidylinositol-(3,4,5)-trisphosphate (PI(3,4,5)P_3_) to generate phosphatidylinositol-(3,4)-bisphosphate (PI(3,4)P_2_). PI(3,4,5)P_3_ is a second messenger lipid that selectively localizes a myriad of signaling proteins (*e.g.*, Akt, BTK, P-Rex1) to the plasma membrane that are critical for cell growth, survival, and development (4, 5, 6). Because PI(3,4,5)P_3_ lipids regulate critical cellular processes, these lipids need to be spatially and temporally regulated for cellular homeostasis. Cells lacking SHIP1 display defects in cell adhesion and migration as a consequence of elevated PI(3,4,5)P_3_ levels (7, 8, 9).

To access its lipid substrate, SHIP1 must localize to the plasma membrane. Antibody-induced activation of the immunoglobulin E receptor (FcεRI) in mast cells revealed that SHIP1 undergoes dynamic cycles of plasma membrane recruitment and dissociation, which leads to the emergent property of cortical oscillations (10). It was also shown that stimulation of either the B-cell receptors or the FcγRIIB receptors enhances SHIP1 plasma membrane localization compared with unstimulated cells (11, 12). Although SHIP1 is recruited to the plasma membrane upon activation of several types of immune cell receptors, the mechanism controlling membrane recruitment and activation is unclear. In particular, it is unclear whether SHIP1 is recruited to the plasma membrane through direct interactions with PI(3,4,5)P_3_ lipids or indirectly by proteins that bind to PI(3,4,5)P_3_. Understanding how each domain of SHIP1 contributes to membrane localization and lipid phosphatase activity will close the gap in knowledge concerning whether SHIP1 directly interacts with activated receptors, newly synthesized PIP lipids, or proteins that bridge these interactions.

SHIP1 contains two proposed lipid-binding domains that flank the central phosphatase domain and have been proposed to regulate membrane localization and catalytic activity (Fig. 1*A*). The Pleckstrin homology–related (PH-R) domain was shown to interact with the substrate of SHIP1, PI(3,4,5)P_3_, whereas the C2 domain reportedly binds to the product of SHIP1, PI(3,4)P_2_ (13, 14, 15). Mutations in the PH-R domain disrupted localization to the phagocytic cup during FcγR-mediated phagocytosis in macrophages but did not affect SHIP1 catalysis (13). This suggests that SHIP1 localization to PI(3,4,5)P_3_-containing membranes is independent of its catalytic domain. Biochemical analysis of the SHIP1 paralog, SHIP2, revealed a structural interface between the phosphatase and C2 domains capable of interdomain communication and allosteric regulation (16). The C2 domain of SHIP2 also reportedly interacts with phosphatidylserine (PS) (16), whereas the SHIP1 C2 domain reportedly binds to PI(3,4)P_2_ (14). Based on sequence homology between SHIP1 and SHIP2, however, both phosphatases are predicted to have similar specificity for PS lipids. Together, these reported lipid interactions suggest a possible mechanism for positive feedback and allosteric activation during SHIP1-mediated dephosphorylation of PI(3,4,5)P_3_. Although this proposed mechanism of SHIP1 is intriguing, there is minimal structural biochemistry data or mutational analysis to corroborate the proposed PIP lipid–binding specificity of SHIP1.

**Figure 1:**
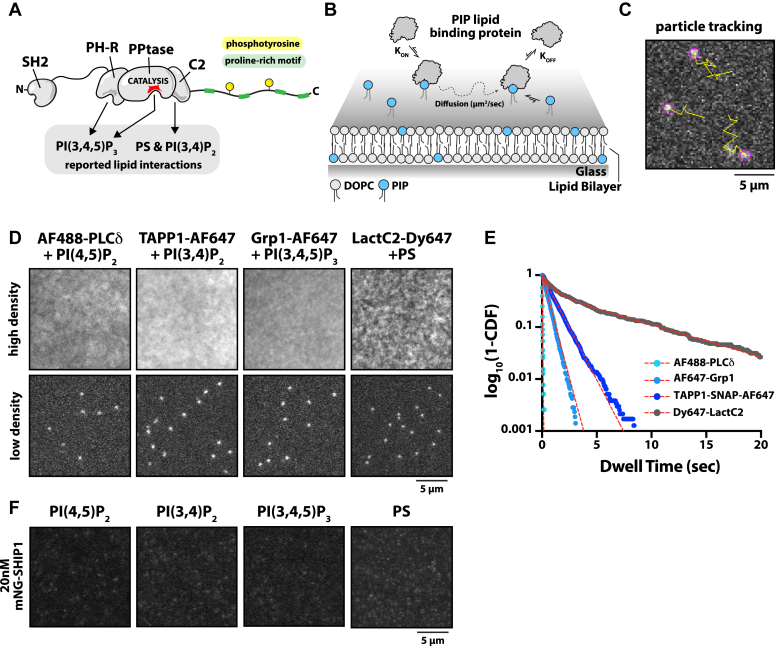
**SHIP1(PH-PP-C2) does not strongly interact with individual PIP lipids *in vitro*.***A*, cartoon diagram showing the reported lipid-binding domains of SHIP1, which include the phosphatase domain and flanking PH-R and C2 domains. *B*, experimental setup for directly visualizing protein–lipid interactions on SLBs using smTIRF-M. *C*, representative image showing single particle detection (*purple circle*) and trajectories (*yellow line*) of three Grp1-AF647 membrane-bound molecules. Image collected in the presence of 1 pM Grp1-AF647. Membrane composition: 2% PI(3,4,5)P_3_ and 98% DOPC. *D*, representative TIRF-M images showing bulk membrane recruitment in the presence of either 20 nM (“high density”) or (“low density”) AF488-PLCδ (250 pM), TAPP1-SNAP-AF647 (1 pM), Grp1-AF647 (1 pM), or LactC2-Dy647 (2 pM) on SLBs containing DOPC lipids plus either 2% PI(4,5)P_2_, 2% PI(3,4)P_2_, 2% PI(3,4,5)P_3_, or 20% DOPS lipids, respectively. *E*, single-molecule dwell time distributions of various PIP lipid–binding domains plotted as dwell time *versus* log_10_(1-cumulative distribution frequency). Curves are fit with a single or double exponential decay curve yielding the following dwell times: AF488-PLCδ (τ_1_ = 24 ± 2 ms), TAPP1-SNAP-AF647 (τ_1_ = 1.02 ± 0.053 s), Grp1-AF647 (τ_1_ = 0.544 ± 0.007 s), or LactC2-Dy647 (τ_1_ = 0.765 ± 0.191 s, τ_2_ = 6.58 ± 0.539 s, α = 0.5 ± 0.04). See Table S1 for statistics. *F*, mNG-SHIP1(PH-PP-C2) does not robustly associated with SLBs containing PIP or PS lipids. Representative images showing bulk membrane recruitment in the presence of 20 nM mNG-SHIP1(PH-PP-C2) on SLBs containing DOPC lipids plus either 2% PI(4,5)P_2_, 2% PI(3,4)P_2_, 2% PI(3,4,5)P_3_, or 20% DOPS, respectively. Note that image intensities were scaled in a similar manner to the top row of images shown in (*C*). DOPC, 1,2-dioleoyl-*sn*-glycero-3-phosphocholine; PIP, phosphatidylinositol phosphate; PI(3,4)P_2,_ phosphatidylinositol-(3,4)-bisphosphate; PI(3,4,5)P_3_, phosphatidylinositol-(3,4,5)-trisphosphate; PS, phosphatidylserine; SHIP1, Src homology 2 domain–containing inositol 5-phosphatase 1; SLB, supported lipid bilayer; smTIRF, single-molecule total internal reflection fluorescence.

Previous biochemical characterization of SHIP1 suggests that the C-terminal domain attenuates 5-phosphatase activity. In particular, a C-terminal truncation was previously shown to increase SHIP1 activity approximately eightfold compared with full-length (FL) SHIP1 (17). Consistent with this result, C-terminal truncation mutants of SHIP2 were also more active than the FL phosphatase (18). A potential mechanism for autoinhibition could be mediated through intramolecular interactions between the N-terminal SH2 domain and C-terminal phosphotyrosine(s). Supporting this model, a peptide derived from the C-terminal region of SHIP1 containing phosphorylated tyrosine residue Y1022 was previously shown to pull down a purified SH2 domain derived from SHIP1 (19). Similar to Src-family tyrosine kinases (20, 21), the N-terminal SH2 domain of SHIP1 could potentially form an intramolecular inhibitory interaction with a C-terminal phosphotyrosine residue (*e.g.*, Y1022). Although Y1022 was shown to be phosphorylated by Src family kinases (22), it remains unclear whether this post-translational modification is responsible for SHIP1 autoinhibition or if it regulates interactions with peripheral membrane–binding proteins in cells. Despite evidence supporting SHIP1 autoinhibition, the mechanism that regulates the formation and relief of intramolecular interactions remains unknown. Understanding the mechanism of autoinhibition could reveal how SHIP1 is recruited to the plasma membrane and the functional consequences of SHIP1 mutants that lack autoinhibitory contacts.

Given the combination of protein and lipid interactions that potentially regulate SHIP1, it remains unclear whether membrane localization of SHIP1 is controlled directly or indirectly by PIP–lipid interactions. To date, no biochemical studies have directly visualized the interactions between purified SHIP1 and proposed PIP lipid–binding partners. Previous experiments have relied on surface plasma resonance (15) and protein lipid overlay assays (13, 14) to measure SHIP1–lipid interactions. These experimental approaches rely on nonfluid lipid monolayers or nitrocellulose membranes to detect interactions between SHIP1 and PIP lipids. Since these methods were used to indirectly visualize SHIP1–lipid interactions, it remains unclear whether SHIP1 molecules dynamically associate and dissociate from lipids. To directly visualize proposed interactions between SHIP1 and different PIP lipid species, we established a single-molecule total internal reflection fluorescence (smTIRF) microscopy assay in combination with supported lipid bilayers (SLBs). Direct visualization of fluorescently labeled SHIP1 revealed very weak lipid-binding specificity compared with several well-established PIP lipid–binding proteins that displayed a range of affinities (*i.e.*, *K*_*D*_ = 0.01–10 μM) (23, 24, 25, 26, 27). In live cells, we also found that the central lipid phosphatase domain of SHIP1 flanked by the two proposed lipid-binding domains transiently associated with the plasma membrane and was nonresponsive to G-protein–coupled receptor–induced changes in PI(3,4,5)P_3_ and PI(3,4)P_2_ levels. Reconstitution of SHIP1–lipid interactions *in vitro* on SLBs revealed that a combination of PS and PI(3,4,5)P_3_ lipids was required to detect very transient membrane dwell times. Biochemical reconstitution of SHIP1 phosphatase activity revealed that the FL enzyme is autoinhibited compared with the central catalytic domain (*i.e.*, PH-PP-C2). Characterization of SHIP1 truncation mutants revealed that both the N-terminal SH2 domain and the disordered C-terminal tail limit SHIP1 phosphatase activity but to different extents. In the presence of an immune receptor–derived tyrosine-phosphorylated peptide added in solution or membrane tethered, SHIP1–lipid phosphatase activity was robustly stimulated. Overall, our results reveal that interactions between SHIP1 and various lipid species are surprisingly weak and likely serve a secondary role following membrane recruitment mediated by SHIP1–protein interactions.

## Results

### SHIP1 does not strongly interact with individual PIP lipids

Flanking the central phosphatase domain, SHIP1 contains two proposed lipid-binding domains (Fig. 1*A*). The PH-R domain (herein referred to as “PH”) was previously shown to interact with the substrate of SHIP1, PI(3,4,5)P_3_ (13), whereas the C2 domain reportedly binds to the product of SHIP1, PI(3,4)P_2_ (14). These lipid interactions are hypothesized to function in concert to regulate SHIP1 membrane docking and lipid phosphatase activity. A major limitation in our understanding of these lipid interactions is that previous experiments utilized binding assays that did not directly visualize dynamic membrane association, bilayer diffusion, and dissociation of SHIP1 (Fig. 1*B*). Observing these behaviors are an essential criterion needed to validate that a proposed lipid-binding domain can autonomously and reversibly interact with a specific lipid.

To assess the role that lipids serve in controlling SHIP1 membrane localization, we established a method to directly visualize the dynamic and reversible membrane-binding interactions using SLBs visualized by TIRF microscopy (Fig. 1, *B* and *C*). We purified and fluorescently labeled a collection of lipid-binding domains derived from LactC2, PLCδ, TAPP1, and Grp1. These domains have well-established interactions with either PS, PI(4,5)P_2_, PI(3,4)P_2_, or PI(3,4,5)P_3,_ respectively (23, 24, 25, 26, 27). Each protein has a *K*_*D*_ in the range of 0.01 to 10 μM, providing a panel of positive controls for directly visualizing various PIP–lipid interactions *in vitro*. When we measured the localization of these lipid-binding domains on SLBs using a solution concentration of 20 nM for each PIP lipid–binding protein, we observed robust membrane recruitment based on the high membrane fluorescence intensity that blanketed the SLB compared with the background signal (Fig. 1*D*). To compare the relative strengths of these protein–lipid interactions, we measured the dwell time of single spatially resolved membrane-binding events (Fig. 1*E* and Movies S1–S4). Consistent with previous *K*_*D*_ measurements based on solution-binding assays, we calculated a range of single-molecule dwell times (τ_1_ = 0.02–5 s) for these lipid-binding proteins (Figs. 1*E* and S1; Table S1).

In order to measure single-molecule interactions between SHIP1 and various phospholipids, we recombinantly expressed and purified an mNeonGreen (mNG)-tagged SHIP1 truncation containing the central phosphatase domain flanked by two lipid-binding domains (*i.e.*, mNG-PH-PP-C2). Compared with the membrane localization of the established lipid-binding domains (Fig. 1, *D* and *E*), we observed no significant membrane association of mNG-SHIP1(PH-PP-C2) on SLBs containing either PS, PI(4,5)P_2_, PI(3,4)P_2_, or PI(3,4,5)P_3_ lipids alone (Fig. 1*F*). Although a small fraction of molecules bound to the SLB, the lack of diffusivity indicated that these rare SHIP1-binding events represented nonspecific interactions with diffraction-limited defects in the SLB. Increasing the frame rate of image acquisition to >80 frames per seconds allowed us to observe low-frequency membrane-binding events that lasted only one frame (12 ms) in the presence of either PS or PI(3,4,5)P_3_ lipids alone. Overall, the mNG-SHIP1(PH-PP-C2) membrane interactions were too transient for us to calculate a characteristic dwell time using any of the membrane compositions described previously.

### Plasma membrane localization of SHIP1(PH-PP-C2) is transient and insensitive to dynamic PI(3,4,5)P_3_ production

**Figure 2:**
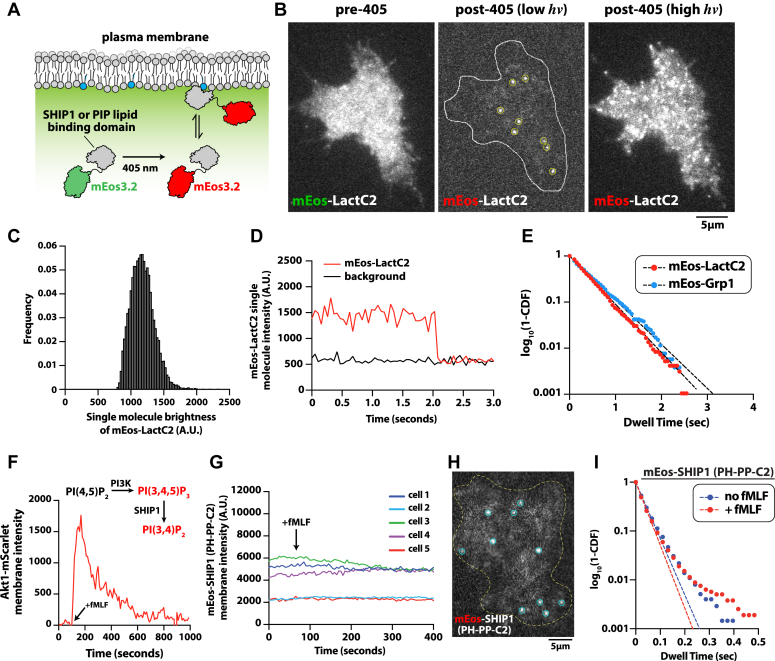
**SHIP1(PH-PP-C2) plasma membrane localization is insensitive to dynamic changes in PI(3,4,5)P**_**3**_**lipid composition.***A*, cartoon diagramming UV-dependent photoconversion and plasma membrane binding of a mEos-tagged cytoplasmic protein in cells. *B*, representative images showing localization of mEos-LactC2 before and after photoconversion with 405 nm UV light (“low” and “high” laser power). Localization of mEos-LactC2 was visualized using smTIRF-M in PLB-985 cells. *C*, molecular brightness distribution of single plasma membrane–localized mEos-LactC2 molecules. *D*, stepwise photobleaching of a single plasma membrane–localized mEos-LactC2 molecule compared with the background fluorescence of the cell. *E*, single-molecule dwell distributions of mEos-LactC2 and mEos3.2-Grp1. Curves were fit to a single exponential decay curve to calculate the following dwell times: mEos-LactC2 (τ_1_ = 371 ± 7 ms, N = 12 cells) and mEos-Grp1 (τ_1_ = 392 ± 5 ms, N = 10 cells). *F*, plot showing the translocation of Akt1-mScarlet to the plasma membrane following stimulation of PLB-985 cells with 10 nM fMLF. *G*, bulk membrane localization of nonphotoconverted mEos-SHIP1(PH-PP-C2) does not change in response to stimulating PLB-985 cells with 10 nM fMLF (*arrow*). *Traces* represent the single cell plasma membrane intensity of mEos-SHIP1(PH-PP-C2) measured by TIRF-M. *H*, representative image showing single particle detection of mNG-SHIP1 in live PLB-985 cells. *I*, single-molecule dwell time distributions of photoconverted mEos-SHIP1(PH-PP-C2) in PLB-985 cells ± 10 nM fMLF plotted as dwell time *versus* log_10_(1-cumulative distribution frequency). Curves are fit with a single exponential decay curves, and both curves yield the following dwell times for mEos-SHIP1(PH-PP-C2): τ_1_ = 38 ± 3 ms (pre-fMLF; N = 4 cells) and τ_1_ = 37 ± 5 ms (post-fMLF; N = 5 cells). A single dwell time was measured for each cell with n = 421 to 4267 molecules tracked per cell. Dwell times are reported as mean ± SD. See Table S1 for statistics. fMLF, *N*-formyl-methionine-leucine-phenylalanine; PI(3,4,5)P_3_, phosphatidylinositol-(3,4,5)-trisphosphate; SHIP1, Src homology 2 domain–containing inositol 5-phosphatase 1; TIRF, total internal reflection fluorescence.

Our *in vitro* characterization of SHIP1 on supported membranes by smTIRF microscopy lacks some of the complexity that exists on the cellular plasma membrane. To determine whether the membrane-binding behavior of SHIP1(PH-PP-C2) is significantly different *in vitro* and *in vivo*, we established conditions to visualize the plasma membrane localization of PIP lipid–binding domains (*i.e.*, Grp1 and LactC2) and SHIP1(PH-PP-C2) in neutrophil-like PLB-985 cells with single-molecule resolution. For these experiments, we tagged Grp1, LactC2, and SHIP1(PH-PP-C2) with a green to red photoconvertible mEos3.2 (referred to as mEos) (Fig. 2*A*). Genes encoding these proteins were transduced into PLB-985 cells using lentivirus, which resulted in a uniform localization pattern across the plasma membrane (Fig. 2*B*). To resolve single-membrane–binding events, we photoconverted a fraction of the mEos-tagged proteins to the more photostable red fluorescent state using a transient pulse of 405 nm light (Fig. 2*B*). The single-molecule brightness distribution and photobleaching analysis indicated that tracked mEos-tagged molecules represented single spatially resolved fluorescent proteins (Fig. 2, *C* and *D*). Single-molecule dwell time analysis of plasma membrane–localized mEos-Grp1 and mEos-LactC2 produced very similar dwell times of 392 ms and 371 ms, respectively (Fig. 2*E*, Movies S5 and S6, Table S1). Given that the dwell times of fluorescently labeled Grp1 and LactC2 were longer in our *in vitro* SLB assay, the *in vivo* dwell times for these lipid-binding proteins likely represent the upper limit for single-molecule track length measured in cells under our experimental conditions. By comparison, mEos-SHIP1(PH-PP-C2) expressed in PLB-985 cells exhibited plasma membrane interactions that were comparatively transient in nature (***τ***_1_ = 38 ms) (Fig. 2*I*, Movie S7, Table S1). Looking at the single-molecule dwell time distributions for mEos-SHIP1(PH-PP-C2), we find that 1 to 2% of all molecules do not fit to the single exponential decay curve. Given the complexity of the cellular plasma membrane, this population of SHIP1 molecules could represent molecules confined in membrane invaginations or part of molecular assemblies that are currently undefined. Notably in live cells, we observed a large fraction of photoconverted mEos-SHIP1(PH-PP-C2) rapidly moving in and out of the TIRF-M focal plane (Movie S7). This population of molecules appears as a diffuse fluorescent cloud near the plasma membrane. This indicates that the vast majority of SHIP1 molecules localizes to the plasma membrane but are unable to persistently engage lipids to produce a spatially resolved and quantifiable dwell time.

To determine whether the membrane-binding dynamics of mEos-SHIP1(PH-PP-C2) could be modulated by acute changes in substrate or product availability at the plasma membrane, we stimulated PLB-985 neutrophil-like cells with a uniform concentration of chemoattractant, *N*-formyl-methionine-leucine-phenylalanine (fMLF). Activation of the formyl peptide receptor with fMLF results in a transient spike in PI(3,4,5)P_3_ and PI(3,4)P_2_ production (28, 29), which we visualized using the dual specificity lipid-binding domain, Akt1-mScarlet (Fig. 2*F*). Following PLB-985 cell activation with 10 nM fMLF, we observed no change in the bulk plasma membrane localization of mNG-SHIP1(PH-PP-C2) (Fig. 2*G*). Similarly, single-molecule dwell time analysis revealed that membrane avidity of mEos-SHIP1(PH-PP-C2) was insensitive to dynamic changes in PI(3,4,5)P_3_ and PI(3,4)P_2_ levels (Fig. 2*I*, Table S1, Movie S7). Consistent with our *in vitro* observations, this result supports an absence of high-affinity interactions between SHIP1(PH-PP-C2) and either PI(3,4,5)P_3_ or PI(3,4)P_2_ lipids *in vivo*.

### SHIP1 catalyzes dephosphorylation of PI(3,4,5)P_3_ with first-order kinetics

**Figure 3:**
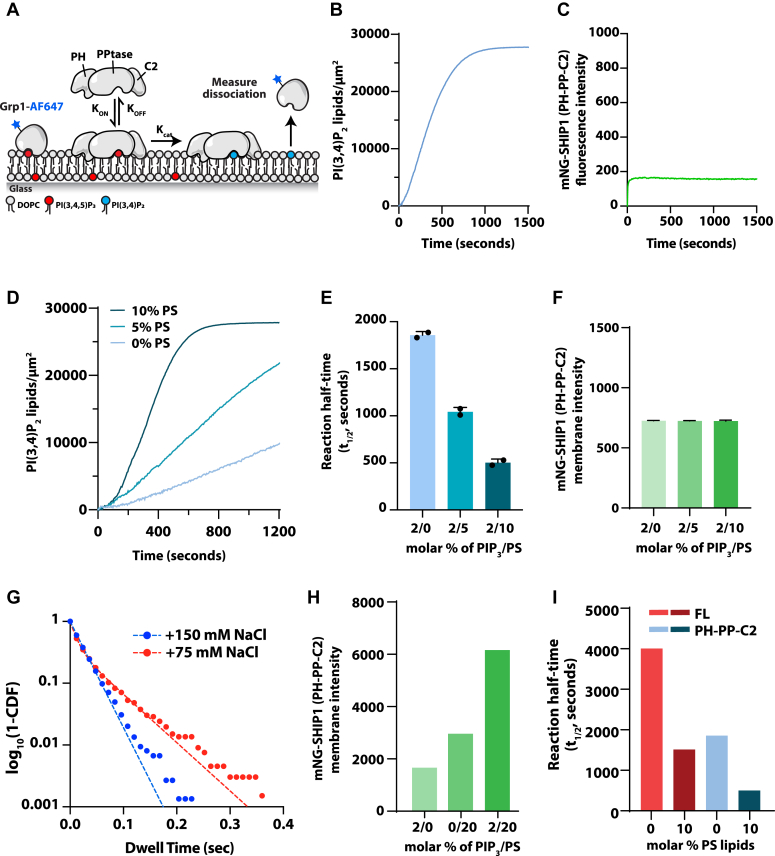
**SHIP1 catalyzes the dephosphorylation of PI(3,4,5)P**_**3**_**with first-order kinetics.***A*, experimental design for measuring SHIP1–lipid phosphatase activity *in vitro* on supported lipid bilayers using TIRF-M. *B*, kinetics of 20 nM SHIP1(PH-PP-C2). PI(3,4,5)P_3_ dephosphorylation measured using 20 nM Grp1-AF647. *C*, bulk membrane localization of 20 nM mNG-SHIP1 during catalysis shown in (*B*). *D*, PS lipids enhance SHIP1 phosphatase activity. Representative kinetics traces of 4 nM mNG-SHIP1 (PH-PPtase-C2 domain) in the absence and presence of PS lipids. Production of PI(3,4)P_2_ was monitored by the presence of 20 nM Grp1-AF647. Initial membrane composition: 88 to 98% DOPC, 0 to 10% PS lipids, and 2% PI(3,4,5)P_3_. *E*, quantification of reaction half time in (*D*). Bars equal mean values (N = 2 reactions per concentration, error = SD). *F*, quantification of bulk localization measured in the presence of 20 nM mNG-SHIP1(PH-PPtase-C2) on SLBs containing 2% PI(3,4,5)P_3_ and 0, 5, 10% PS lipids (N = 10 fluorescent intensity measurements per membrane, error = SD). *G*, single-molecule dwell time measured in the presence of 200 pM mNG-SHIP1(PH-PPtase-C2). Data plotted as dwell time *versus* log_10_(1-cumulative distribution frequency). Curves fit with a single or double exponential decay curve yielding the following dwell times: 150 mM NaCl buffer (τ_1_ = 25 ± 1 ms) or 75 mM NaCl buffer (τ_1_ = 9 ± 2 ms, τ_2_ = 56 ± 7 ms, α = 0.44 ± 0.13). Membrane composition: 88% DOPC, 2% PI(3,4,5)P_3_, 20% PS. *H*, quantification of bulk localization measured in the presence of 20 nM mNG-SHIP1(PH-PPtase-C2) on SLBs containing 2% PI(3,4,5)P_3_ and 0-20% PS lipids. *I*, full-length SHIP1 is stimulated by PS lipids but exhibits lower activity compared with mNG-SHIP1(PH-PPtase-C2). Reaction half times comparing phosphatase activity in the presence of 4 nM full-length mNG-SHIP1 or 4 nM mNG-SHIP1(PH-PPtase-C2). Membrane composition: 88 to 98% DOPC, 2% PI(3,4,5)P_3_, ±10% PS. See Table S1 for statistics. DOPC, 1,2-dioleoyl-*sn*-glycero-3-phosphocholine; PI(3,4,5)P_3_, phosphatidylinositol-(3,4,5)-trisphosphate; PS, phosphatidylserine; SHIP1, Src homology 2 domain–containing inositol 5-phosphatase 1; SLB, supported lipid bilayer; TIRF, total internal reflection fluorescence.

Based on reported lipid interactions, researchers have hypothesized that SHIP1 catalyzes the dephosphorylation of PI(3,4,5)P_3_ with a positive feedback loop based on interactions with both substrate and product (13, 14, 15). However, no positive feedback mechanism has been experimentally reconstituted using purified SHIP1 to drive the dephosphorylation of PI(3,4,5)P_3_. To measure potentially nonlinear reaction kinetics, we developed an *in vitro* assay to simultaneously visualize SHIP1 membrane-binding and lipid phosphatase activity using TIRF microscopy. For these experiments, we used both fluorescently labeled Grp1 and TAPP1 PH domains to monitor changes in the membrane abundance of PI(3,4,5)P_3_ and PI(3,4)P_2_ (Fig. S2, *A*–*C*), respectively. Using fluorescently labeled TAPP1, we found that the interaction with PI(3,4)P_2_ was quite strong and resulted in continual membrane binding even after SHIP1-mediated dephosphorylation of PI(3,4,5)P_3_. This made quantification of reaction completion times ambiguous under experimental conditions that utilize varying concentrations of PS lipids. For this reason, we primarily used the Grp1-AF647 sensor to monitor SHIP1-mediated dephosphorylation of PI(3,4,5)P_3_ on supported membranes (Figs. 3*A* and S2, *D*–*F*). To display kinetic traces in terms of product formation, we inverted the Grp1 membrane dissociation curves to show the time-dependent increase in SHIP1 product formation (Fig. 3*B*). Independent of how data were plotted, kinetic traces were hyperbolic shaped (Figs. 3*B* and S2). Consistent with SHIP1-catalyzing reactions with simple first-order kinetics, we observed no reciprocal regulation–linking changes in PI(3,4,5)P_3_ or PI(3,4)P_2_ density to membrane localization of SHIP1 (Fig. 3*C*). If there was any reciprocal regulation resulting from lipid interactions, albeit weak, SHIP1 membrane recruitment curves would be more sigmoidal in shape and increase following the production of PI(3,4)P_2_ lipids. However, the membrane fluorescence intensity of mNG-SHIP1(PH-PP-C2) remained constant throughout the course of the reaction (Fig. 3*C*). This was consistent with our inability to detect interactions between mNG-SHIP1(PH-PP-C2) and either PI(3,4,5)P_3_ or PI(3,4)P_2_ lipids by smTIRF (Fig. 1, *E* and *F*).

PS lipids have been reported to bind to the C2 domain of SHIP2 and allosterically regulate its phosphatase activity (16). Consistent with this model, we measured enhanced SHIP1 phosphatase activity over a range of PS lipid densities (Fig. 3, *D* and *E*). However, the increase in activity was not correlated with a dramatic change in bulk membrane localization of mNG-SHIP1(PH-PP-C2) (Fig. 3*F*). PS-dependent enhancements in activity without any associated enhancement in bulk membrane localization were surprising but consistent with a mechanism of allostery or regulation of the SHIP1 membrane docking orientation. Next, we returned to single-molecule dwell time analysis of mNG-SHIP1(PH-PP-C2) using supported membranes containing a mixture of 2% PI(3,4,5)P_3_ and 20% PS lipids. Under these conditions, we detected very transient mNG-SHIP1(PH-PP-C2) membrane-binding dynamics (***τ***_1_ = 25 ms, Fig. 3*G*, Table S1, Movie S8). These single-molecule dwell times were nearly identical to our measurements of mEos-SHIP1(PH-PP-C2) binding to the plasma membrane in neutrophil-like cells (***τ***_1_ = 38 ms, Fig. 2*I*).

To determine if the mNG tag sterically hinders SHIP1(PH-PP-C2) from binding membranes containing 2% PI(3,4,5)P_3_ and 20% PS lipids, we measured the single-molecule dwell times for SHIP1(PH-PP-C2) that was sortase labeled at the N-terminal amine with AF488. The dwell times measured in the presence of AF488-SHIP1(PH-PP-C2) (***τ***_1_ = 24 ms) were indistinguishable from mNG-SHIP1(PH-PP-C2) (Fig. S3, Table S1). We also observed very transient mNG-SHIP1(PH-PP-C2) lipid interactions in the presence of NaCl- and KCl-containing buffers (Fig. S4, *A* and *B*) as well as membranes containing either phosphatidylcholine or phosphatidylethanolamine lipids (Fig. S4, *C* and *D*). To strengthen the weak interactions observed between mNG-SHIP1(PH-PP-C2), PI(3,4,5)P_3_, and PS lipids, we reduced the buffer ionic strength (75 mM NaCl), and repeated our dwell time analysis. Under these conditions, membrane-associated mNG-SHIP1(PH-PP-C2) changed to display two characteristic dwell times (***τ***_1_ = 9 ms, ***τ***_2_ = 56 ms, ⍺ = 44%, Fig. 3*G* and Table S1). In the presence of lower ionic strength buffer, we also observed a modest increase in bulk membrane localization of mNG-SHIP1(PH-PP-C2) that was dependent on PI(3,4,5)P_3_ and PS lipids (Fig. 3*H*).

Next, we aimed to determine if PS lipids could similarly increase phosphatase activity of FL SHIP1 (1–1188 amino acids [aa]). Although we found that PS lipids enhanced the activity of FL SHIP1, the overall activity was still reduced compared with SHIP1(PH-PP-C2) (Fig. 3*I*). Although PS lipids are considered allosteric regulators of SHIP1, this result suggests that the domains flanking PH-PP-C2 suppress the catalytic activity of SHIP1.

### FL SHIP1 is autoinhibited by the N-terminal SH2 domain

**Figure 4:**
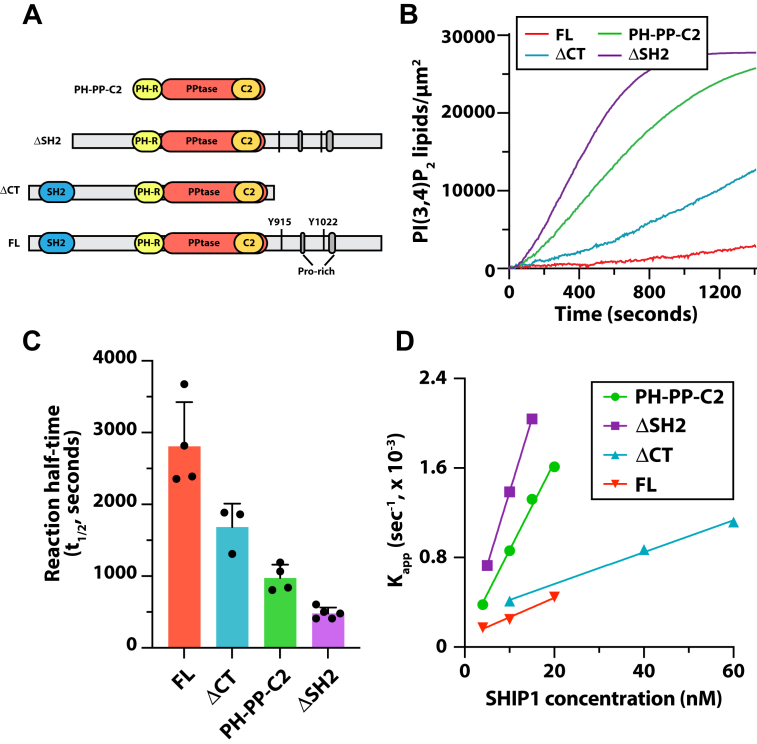
**Full-length (FL) SHIP1 is autoinhibited by the N-terminal SH2 domain.***A*, domain organization of FL SHIP1 and truncation mutants used in supported lipid bilayer TIRF-M assays. *B*, kinetic traces showing the dephosphorylation of PI(3,4,5)P_3_ in the presence 10 nM mNG-SHIP1 (1–1188 aa), mNG-SHIP1(PH-PPtase-C2), mNG-SHIP1(ΔSH2), and mNG-SHIP1(ΔCT). *C*, quantification of reaction half times measured in the presence of 10 nM SHIP1, FL, and truncation mutants. Bars equal mean values (N = 4–5 technical replicates, error = SD). *D*, plot shows the relationship between SHIP1 concentration and phosphatase activity. Comparing mNG-SHIP1 (1–1188 aa), mNG-SHIP1(PH-PPtase-C2), mNG-SHIP1(ΔSH2), and mNG-SHIP1(ΔCT). *B*–*D*, initial membrane composition: 98% DOPC and 2% PI(3,4,5)P_3_. DOPC, 1,2-dioleoyl-*sn*-glycero-3-phosphocholine; PI(3,4,5)P_3_, phosphatidylinositol-(3,4,5)-trisphosphate; SH2, Src homology 2; SHIP1, Src homology 2 domain–containing inositol 5-phosphatase 1; TIRF, total internal reflection fluorescence.

It was previously demonstrated that truncating the C terminus of SHIP1 enhances phosphatase activity of FL SHIP1, suggesting the C terminus negatively regulates phosphatase activity (17). By contrast, studies using SHIP2 immunoprecipitated from cell lysate found that the N-terminal SH2 domain in conjunction with the C terminus inhibits SHIP2 activity (18). To investigate potential mechanisms of SHIP1 autoinhibition, we purified FL recombinant SHIP1, an N-terminal truncation mutant (ΔSH2), and a C-terminal truncation mutant (ΔCT) using baculovirus expression in insect cells (Fig. 4*A*). Using our SLB TIRF assay, we measured the phosphatase activity of FL SHIP1 and truncation mutants. These experiments revealed that FL SHIP1 has reduced activity compared with SHIP1(PH-PP-C2) (Fig. 4*B*). Although it was previously demonstrated that truncating the C terminus of SHIP1 increased activity ninefold (17), removal of this region only increased SHIP1 activity 1.8-fold compared with the FL protein (Fig. 4, *B*–*D*). The C terminus of SHIP1 has two NPxY motifs that can be phosphorylated by Src family kinases. However, the increase in activity observed for the ΔCT mutant is unlikely the result of these post-translational modifications. According to Western blot, SHIP1 purified from insect cells lack any detectable tyrosine phosphorylation (Fig. S4*D*). In addition, incubation of FL SHIP1 with the promiscuous tyrosine phosphatase, YopH, did not modulate phosphatase activity (Fig. S4*E*). Biochemical characterization of the SHIP2 isoform found that the N-terminal SH2 domain in conjunction with the C terminus inhibits SHIP2 activity (18). In our experiments, removal of the N-terminal SH2 domain of SHIP1 alone yielded an activity curve nearly identical to our PH-PP-C2 truncation mutant, suggesting that SHIP1 autoinhibition is largely dependent on the SH2 domain (Fig. 4, *B*–*D*).

### Membrane recruitment and activation of SHIP1 by a phosphotyrosine peptide

**Figure 5:**
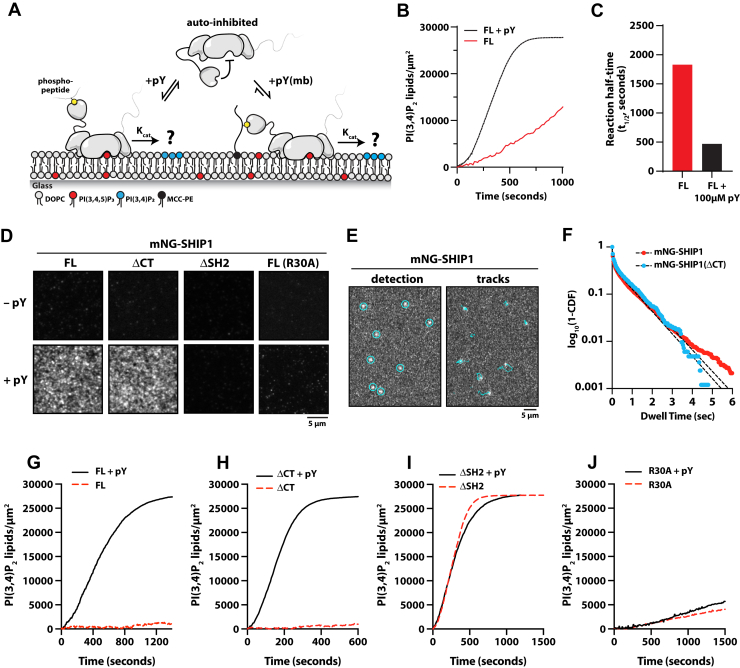
**Phosphotyrosine peptides promote membrane recruitment and activation of SHIP1.***A*, experimental design for measuring SHIP1 phosphatase activity in the presence of phosphotyrosine peptides derived from ITIM motif of the FcγRIIB receptor (pY-ITIM) in solution or membrane conjugated. *B*, 100 μM pY-ITIM in solution stimulates the phosphatase activity of 20 nM full-length (FL) SHIP. Initial membrane composition: 96% DOPC, 2% PI(3,4,5)P_3_, and 2% MCC-PE (quenched/nonreactive). *C*, quantification of reaction half times in (*B*). *D*, representative TIRF-M images showing localization of 5 nM mNG-SHIP1(FL, 1–1188 aa), mNG-SHIP1(ΔCT), mNG-SHIP1(ΔSH2), and mNG-SHIP1(R30A) in the presence of pY-ITIM conjugated to an SLB. *E*, representative TIRF-M images showing the detection and tracking of mNG-SHIP1 (1–1188 aa) bound to a pY membrane. *F*, single-molecule dwell time distribution measured in the presence of 20 pM mNG-SHIP1(1–1188 aa) or 20 pM mNG-SHIP1(ΔCT) on SLBs containing membrane-conjugated pY-ITIM peptide. Curves fit with a double exponential decay curve (*black dashed line*) yielding the following dwell times: mNG-SHIP1 (1–1188 aa) (τ_1_ = 54 ± 4 ms, τ_2_ = 872 ± 82 ms, α = 0.62) and mNG-SHIP1(ΔCT) (τ_1_ = 55 ± 5 ms, τ_2_ = 941 ± 17 ms, α = 0.61). Dwell times are reported as mean ± SD. See Table S1 for statistics. *G*–*J*, SHIP1 phosphatase activity measured in the absence and presence of the membrane-conjugated pY-ITIM peptide. Phosphatase activity was measured using the following SHIP1 solution concentrations: (*G*) 0.1 nM mNG-SHIP1(FL, 1–1188 aa), (*H*) 0.1 nM mNG-SHIP1 (ΔCT), (*I*) 5 nM mNG-SHIP1(ΔSH2), (*J*) 5 nM mNG-SHIP1(R30A). Production of PI(3,4)P_2_ was monitored in the presence of 20 nM Grp1-AF647. Plots were inverted to display the production of PI(3,4)P_2_, rather than the depletion of PI(3,4,5)P_3_. *D*–*J*, membrane composition: 96% DOPC, 2% PI(3,4,5)P_3_, 2% MCC-PE-(pY conjugated). DOPC, 1,2-dioleoyl-*sn*-glycero-3-phosphocholine; ITIM, immunoreceptor tyrosine inhibitory motif; MCC-PE, 1,2-dioleoyl-*sn*-glycero-3-phosphoethanolamine-*N*-[4-(p-maleimidomethyl)cyclohexane-carboxamide; PI(3,4)P_2_, phosphatidylinositol-(3,4)-bisphosphate; PI(3,4,5)P_3_, phosphatidylinositol-(3,4,5)-trisphosphate; pY, tyrosine-phosphorylated motif; SHIP1, Src homology 2 domain–containing inositol 5-phosphatase 1; SLB, supported lipid bilayer; TIRF, total internal reflection fluorescence.

During cell signaling, SH2 domains play a critical role regulating membrane recruitment of cytoplasmic proteins to immune receptors that contain tyrosine-phosphorylated motifs (pY) (22, 30). Because truncating the SH2 domain of SHIP1 relieved autoinhibition, we hypothesized that phosphopeptide interactions with the SH2 domain could activate SHIP1 by relieving autoinhibition and enhancing membrane localization (Fig. 5*A*). To decipher this mechanism, we derived a peptide from the immunoreceptor tyrosine inhibitory motif (ITIM) of the FcγRIIB receptor containing a single phosphotyrosine residue (pY-ITIM). SHIP1 is reported to be critical for the inhibitory function of FcγRIIB (31), and this peptide was previously shown to interact with the SH2 domain of SHIP1 in a pull-down assay (19). In solution, the pY-ITIM peptide enhanced the activity of FL SHIP1 nearly fivefold (Fig. 5, *B* and *C*).

*In vivo*, SHIP1 is recruited to the plasma membrane by pY-ITIM following receptor activation in B-cells (22). To determine how membrane-anchored pY-ITIM regulates SHIP1 membrane recruitment and activation, we covalently attached the pY-ITIM peptide to a cysteine-reactive maleimide lipid that was incorporated into supported membranes containing PI(3,4,5)P_3_ lipids (Fig. 5*D*). We found that pY-ITIM covalently coupled to lipids robustly recruited and activated SHIP1 compared with experiments performed with the pY-ITIM peptide in solution (Fig. 5, *D* and *G*). We also measured rapid activation of the partially autoinhibited C-terminal truncation mutant (ΔCT) and localization to SLBs (Fig. 5, *D* and *H*). Consistent with our bulk membrane recruitment experiments (Fig. 5*D*), we measured an average dwell time of ∼0.4 s for both FL mNG-SHIP1 and mNG-SHIP1(ΔCT) in the presence of membrane-conjugated pY-ITIM peptide (Fig. 5, *E* and *F*, Table S1, Movie S9). By contrast, we did not observe pY-dependent localization of the N-terminal truncation mutant (ΔSH2) or R30A mutation (Fig. 5*D*), which are both predicted to eliminate the ability to bind to pY peptides. The activity of SHIP1 (ΔSH2) was the same when measured in the absence and presence of the membrane-conjugated pY-ITIM peptide (Fig. 5*I*). By contrast, the SHIP1 (R30A) mutant retained autoinhibition and was insensitive to pY-ITIM activation (Fig. 5*J*). The lack of activity observed for SHIP1 (R30A) further supports a model in which autoinhibition is independent of an intramolecular interaction between the SH2 domain and post-translational modification (*i.e.*, tyrosine phosphorylation). Together, these results further support an autoinhibitory mechanism mediated by the N-terminal SH2 domain and demonstrates that receptor-derived ITIMs can both membrane recruit and stimulate SHIP1 activity.

## Discussion

### SHIP1–lipid-binding specificity

SHIP1 is a multidomain protein with two proposed lipid-binding domains considered important for membrane localization and activity (13, 14) (Fig. 1*A*). Because of a lack of structural data and mutational analysis of these domains, it has been difficult to define the molecular basis of the proposed SHIP1–lipid interactions. To gain insight about the nature of SHIP1–lipid-binding specificity, we directly visualized membrane association and dissociation dynamics of fluorescently labeled SHIP1 on SLBs using smTIRF-M. Despite reports of PIP–lipid interactions being mediated by both the PH-R (13) and C2 domains (14), our *in vitro* single-molecule dwell time analysis indicates that SHIP1 does not strongly associate with PI(4,5)P_2_, PI(3,4)P_2_, PI(3,4,5)P_3_, or PS lipids that are individually incorporated into supported membranes (Fig. 1*F*). Based on the reported affinity that SHIP1 has for PI(3,4)P_2_ (*K*_*D*_ = 6 nM) and PI(3,4,5)P_3_ (*K*_*D*_ = 1 nM) (15), we expected to observe single-molecule dwell times greater than 10 s (*k*_*OFF*_ = 0.1 s^−1^). This estimate assumes that SHIP1 membrane association is diffusion limited (*k*_*ON*_ = 10 μM^−1^ s^−1^). Based on the proposed reciprocal regulation between SHIP1–lipid and PIP–lipid interactions, we also expected SHIP1 localization to be cooperative in nature and for activity traces to display positive feedback based on product recognition. Instead, SHIP1 catalyzed PI(3,4,5)P_3_ dephosphorylation with simple first-order kinetics and displayed no enhanced membrane recruitment over the course of any phosphatase-catalyzed reaction. Overall, the lipid-binding interactions we observed for SHIP1 were extremely transient in nature and inconsistent with the specificity and nanomolar affinities previously measured using surface plasma resonance, protein lipid overlay assays, and NMR spectroscopy (13, 14, 15).

We established conditions that enabled the detection of transient SHIP1–lipid interactions on supported membranes containing a mixture of 2% PI(3,4,5)P_3_ and 20% PS (***τ***_1_ = 25 ms, *K*_*OFF*_ = 40 s^−1^). The dwell times we measured over a variety of buffer and lipid compositions (Fig. S4) were similar to those we measured for mEos-SHIP1(PH-PP-C2) interacting with the plasma membrane in human neutrophils (Fig. 2*I*). Since both PI(3,4,5)P_3_ and PS are required to detect a only very transient interaction, the individual lipid interactions must have very weak affinities (*K*_*D*_ > 10 μM). This is consistent with our estimated *K*_*D*_ of 90 μM for the interaction between the SHIP1 active site and PI(3,4,5)P_3_, which we calculated based on a previously measured *K*_*M*_ (94 μM) and *k*_cat_ (8 s^−1^) (16).

### SHIP1 activation by PS

The C2 domain of the SHIP1 paralog, SHIP2, reportedly interacts with PS, which is proposed to allosterically stimulate phosphatase activity (16). Despite including up to 20% PS lipids in our TIRF-M-supported membrane experiments, we did not observe a strong enhancement in the bulk membrane recruitment of mNG-SHIP1(PH-PP-C2). PS lipids did, however, stimulate the lipid phosphatase activity of SHIP1(PH-PP-C2), consistent with the reported mechanism of allosteric regulation for SHIP2 (16). Similarly, we observed a PS-dependent increase in FL SHIP1 activation. However, the overall activity was substantially lower compared with the SHIP1(PH-PP-C2) protein. This suggests that FL SHIP1 is autoinhibited by a mechanism that is partially resistant to PS-mediated activation. Considering that the plasma membrane contains 10 to 20% PS (32), FL SHIP1 would be less susceptible to spurious activation, which would help preserve cellular PI(3,4,5)P_3_ and PI(3,4)P_2_ lipid homeostasis. Based on our single-molecule dwell time analysis of mNG-SHIP1(PH-PP-C2), the presence of both PS and PI(3,4,5)P_3_ lipids synergistically enhances membrane binding. The transient interaction between the C2 domain and PS lipids could help orient the phosphatase domain on the membrane in a manner that makes SHIP1 more catalytically efficient. Looking at sequence homology between SHIP1 and SHIP2, we find that the phosphatase domains share 65% identity, whereas the C2 domains share 43% homology. Although PS-dependent allosteric activation of SHIP1 is potentially mediated by the phosphatase and C2 domain interface, like has been described for SHIP2 (16), the molecular basis remains unclear.

### Mechanism of SHIP1 autoinhibition

We performed a mutational analysis of FL SHIP1 to determine which domains suppress lipid phosphatase activity. We found that deletion of the disordered C terminus partially activates SHIP1, whereas deletion of the N-terminal SH2 domain fully activates the enzyme. Because the C terminus of SHIP1 (892–1188 aa) is predicted to be disordered, it remains unclear whether this region forms intramolecular contacts with a specific folded domain of SHIP1. Alternatively, the extended disordered C-terminal domain of SHIP1 could sterically hinder substrate binding. This type of mechanism has been reported for the guanine nucleotide exchange factor Son of Sevenless (SOS), which similarly contains an extended disordered C-terminal domain and multiple layers of autoinhibition (33). Based on biochemical analysis of SOS-dependent activation of Ras GTPase, researchers previously reported that the presence of the unstructured polyproline domain attenuated SOS guanine nucleotide exchange factor activity by reducing the association rate constant (*k*_*ON*_) (33). To date, however, no specific intramolecular interactions have been identified to regulate the C-terminal–dependent autoinhibition of either SOS or SHIP1 activity.

Since the N and C terminus both contribute to autoinhibition, we initially hypothesized that SHIP1 was regulated by intramolecular interactions between the SH2 domain and tyrosine-phosphorylated residues located in the C terminus. By this mechanism, SHIP1 could adopt an autoinhibited confirmation similar to an Src-family nonreceptor tyrosine kinase (20, 21). Suggestive of this model, the SH2 domain of SHIP1 was previously shown to interact with an isolated peptide containing phosphorylated Y1022 derived from SHIP1 (19). However, since these interactions were reconstituted using minimal fragments, it is unclear whether the SH2 domain and the phospho-Y1022 peptide can form an intramolecular interaction in the context of FL SHIP1. Ultimately, determining the exact intramolecular interactions that regulate SHIP1 autoinhibition will require combining structural biochemistry and mutational analysis.

In our investigation of SHIP1 autoinhibition, we found that incubating SHIP1 with the promiscuous bacterial tyrosine phosphatase denoted YopH (34) did not relieve autoinhibition. Based on Western blot analysis, insect cell purified SHIP1 lacks tyrosine phosphorylation (Fig. S4). In addition, the SHIP1 (R30A) mutant retained autoinhibition (Fig. 5*J*), suggesting that the SH2 domain does not interact with a tyrosine-phosphorylated residue to regulate autoinhibition of SHIP1. In the context of immune cell signaling, the C-terminal NPxY motifs of SHIP1 could be tyrosine phosphorylated. As a result, SHIP1 could adopt autoinhibited conformations that are mediated by intramolecular head-to-tail interactions or intermolecular daisy-chain structures. These molecular assemblies could modulate SHIP1 localization and activity in different signaling contexts. Although we were able to drive the phosphorylation of SHIP1 *in vitro* using c-Src kinase (Fig. S4*D*), it is unclear how phosphorylation modulates membrane recruitment and activity of SHIP1.

In considering potential mechanisms for SHIP1 autoinhibition, we can draw parallels to our structural understanding of autoinhibited class 1A PI3K (35). The regulatory subunit of PI3K contains two SH2 domains that each form intermolecular contacts with the catalytic subunit of PI3K. These interactions occur independently of a tyrosine phosphopeptide interaction. Similar to SHIP1, the SH2 domains of class I PI3Ks bind phosphorylated receptors, which releases autoinhibition to activate PI3K (35). Like PI3K, mutating residue R30A in SH2 domain of SHIP1 abolishes pY binding without disrupting autoinhibition. This supports that SHIP1 autoinhibition is mediated by the SH2 domain, but these interactions are distal to the pY-binding pocket.

### Mechanisms controlling SHIP1 membrane localization in cells

The production of PI(3,4,5)P_3_ lipids during immune cell signaling has been shown to enhance plasma membrane localization of FL SHIP1 (13). However, it has been unclear whether this response is regulated by a direct SHIP1-PI(3,4,5)P_3_ interaction or indirectly mediated by peripheral membrane–binding proteins that bind to PI(3,4,5)P_3_ lipids. Single-molecule visualization of SHIP1 in cells using photoconvertible mEos3.2 revealed that a small fraction of mEos-SHIP1(PH-PP-C2) molecules transiently localize to the plasma membrane. The membrane-binding dynamics of mEos-SHIP1(PH-PP-C2) measured in cells were similarly transient to those measured on SLBs containing 2% PI(3,4,5)P_3_ and 20% phosphatidylserine. Consistent with membrane localization of purified mNG-SHIP1(PH-PP-C2) being insensitive to changes in PIP lipid composition *in vitro*, acute changes in PI(3,4,5)P_3_ levels did not modulate the bulk or single-molecule membrane-binding dynamics of SHIP1 in immune cells. Overall, the apparent lack of high-affinity PS or PIP lipid interactions described here suggests that SHIP1 membrane localization in cells is dominated by interactions with peripheral membrane proteins.

SHIP1 contains a variety of protein interaction domains and motifs that can potentially modulate its membrane localization and phosphatase activity in living cells. In the context of B-cell receptor signaling, SHIP1 is recruited by the ITIM of the inhibitory FcγRIIB receptor to downregulate this signaling process (31). The interaction of SHIP1 with the FcγRIIB receptor is dependent on its SH2 domain and tyrosine phosphorylation of the ITIM (22). Our *in vitro* reconstitution demonstrates for the first time that membrane-tethered phosphotyrosine peptides (*i.e.*, pY-ITIM) can robustly recruit and activate SHIP1 on PI(3,4,5)P_3_-containing membranes. The data presented here also support a model in which SHIP1 activity is largely regulated by its N-terminal SH2 domain. We measured a fivefold increase in activity because of solution pY relieving SHIP1 autoinhibition. In contrast, when we conjugated pY to an SLB and recruited SHIP1, we observe the same level of phosphatase activity using a 40-fold lower concentration of SHIP1. Together, these data suggest that enhanced membrane localization plays the most significant role in stimulating SHIP1 activity. Although the C terminus of SHIP1 has numerous protein interaction motifs that can potentially localize SHIP1 to the plasma membrane, the molecular basis of those interactions has not been elucidated. By filling the gap in knowledge concerning how SHIP1 activity and membrane localization is regulated, in the future, we can better understand how cells control the strength and duration of PI(3,4,5)P_3_-signaling events. Given that SHIP1 is implicated in several diseases, including cancer (36, 37, 38, 39, 40, 41, 42), the described regulatory mechanisms provide new insight about the cellular function of SHIP1.

## Experimental procedures

### Molecular biology

Gene coding for human phospholipase C-delta pleckstrin homology domain (11–140 aa; PH domain, accession no.: P51178.2) was synthesized by GeneArt (Invitrogen) as a codon-optimized open reading frame. The gene coding for the human tandem PH domain–containing protein-1 PH domain (182–303 aa; UniProt accession no.: Q9HB21, *PKHA1*) was cloned using mKate2-P2A-APEX2-TAPP1-PH, a gift from Rob Parton (Addgene plasmid #67662) (43). The gene coding for the lactadherin-C2 PS lipid sensor was cloned from LactC2-GFP, a gift from Sergio Grinstein (Addgene plasmid #22852) (23). The lactadherin-C2 domain was originally cloned from the *Bos taurus* (bovine) gene called *MFGE8* (UniProt accession no.: Q95114). Genes encoding human *CYTH3*/Grp1 (1–400 aa) and human SHIP1 (or INPP5D) were purchased as complementary DNA clones from Horizon (PerkinElmer), formerly known as Open Biosystems and Dharmacon. Lentiviral packing vectors were purchased from Addgene. The psPAX2 plasmid was a gift from Didier Trono (Addgene plasmid #12260). The pVSV-G plasmid was a gift from Akitsu Hotta (Addgene plasmid #138479) (44). Bacterial expression vectors encoding YopH (1–468 aa) and chicken c-Src kinase (his_6_-TEV-Src [251–533 aa]) were provided by the laboratory of John Kuriyan (University of California at Berkeley) (45). Gene fragments were subcloned into bacterial and baculovirus protein expression vectors containing coding sequences with different solubility and affinity tags. SHIP1 was cloned into a modified FAST Bac1 vector using Gibson Assembly. The complete open reading frame of all vectors used in this study was sequenced by Genewiz to ensure the plasmids lacked deleterious mutations. Each protein expression construct was screened for optimal yield and solubility in either bacteria (BL21(DE3) Star, Rosetta, etc.) or *Spodoptera frugiperda* (Sf9) insect cells.

### Generation of baculovirus for recombinant protein expression

BACMID DNA was generated by transforming FASTBac1 plasmids into DH10 Bac cells. Positive clones were isolated based on blue–white colony selection, and BACMID DNA was purified from bacteria as previously described (46). To generate baculovirus, 1 × 10^6^ *S. frugiperda* (Sf9) insect cells were transfected with 5 to 7 μg BACMID DNA plus 4 μl Fugene (ThermoFisher; catalog no.: 10362100) in 2 ml of ESF 921 Serum-Free Insect Cell Culture media (Expression Systems; catalog no.: 96-001-01). As previously described, the viral titer was amplified from 2 ml to 100 ml of the course of 2 weeks (46). The final viral titer was sterilized using a 0.22 μm polyethersulfone filter (Corning; catalog no.: 431153) and stored at 4 °C. For optimal protein expression, baculovirus was used within 3 months.

### Protein purification

#### SHIP1 (FL, ΔCT, ΔSH2, and R30A)

The coding sequence of FL human SHIP1 (1–1188 aa) was cloned into a modified FastBac1 vector containing an N-terminal his6-TEV or his6-TEV-mNG fusion. High Five insect cells were infected with baculovirus using an optimized multiplicity of infection, typically 2% v/v, which we empirically determined based on small-scale test expressions (25–50 ml culture). Infected cells were typically grown for 48 h at 27 °C in ESF 921 Serum-Free Insect Cell Culture medium. Cells were harvested by centrifugation, transferred to 50 ml tubes by washing with 1× PBS (pH 7.2), pelleted, and resuspended in 10 ml of 1× PBS (pH 7.2), 10% glycerol, and 2× protease inhibitor cocktail (one Sigma protease inhibitor tablet per 50 ml of buffer) and then stored in the −80 °C freezer. For purification, frozen cell pellets were thawed in an ambient water bath and lysed into buffer containing 30 mM Tris (pH 8.0), 10 mM imidazole, 400 mM NaCl, 1 mM PMSF, 2 mM beta-mercaptoethanol (BME), Sigma protease inhibitor cocktail EDTA-free per 100 ml lysis buffer, and 100 μg/ml DNase using a dounce homogenizer. Lysate was then centrifuged at 37,000 rpm (140,000*g*) for 60 min in a Beckman Ti70 rotor at 4 °C. High-speed supernatant (HSS) was then batch bound to 5 ml of Ni–NTA Agarose (Qiagen; catalog no.: 30230) resin at 4 °C for 2 h stirring in a beaker. The resin and HSS was collected in 50 ml tubes, centrifuged, and washed with buffer containing 20 mM Tris (pH 8.0), 30 mM imidazole, 300 to 400 mM NaCl, 5% glycerol, and 2 mM BME before being transferred to gravity flow column. Ni–NTA resin with bound his6-TEV-mNG-SHIP1 was then washed with an additional 100 ml of 20 mM Tris (pH 8.0), 30 mM imidazole, 300 to 400 mM NaCl, 5% glycerol, and 2 mM BME buffer and then eluted into buffer containing 300 mM imidazole. Peak fractions were pooled and desalted using a G25 Sephadex desalting column (GE Healthcare/Cytiva) equilibrated in 20 mM Tris (pH 8.0), 100 mM NaCl, and 1 mM Tris(2-carboxyethyl)phosphine (TCEP) buffer. Any precipitation was removed *via* 0.22 μm syringe filtration. Clarified SHIP1 fractions were then bound to a MonoQ anion exchange column (GE Healthcare/Cytiva) equilibrated in 20 mM Tris (pH 8.0), 100 mM NaCl, and 1 mM TCEP buffer. Proteins were resolved over a 10 to 100% linear gradient (0.1–1 M NaCl, 30 min, 1.5 ml/min flow rate). His6-TEV-mNG-SHIP1 elutes in the presence of 200 to 400 mM NaCl. Peak fractions containing SHIP1 were pooled, incubated with 400 μl of 243 μM his6-TEV protease (S291V) with poly-R tail for ∼12 to 16 h at 4 °C, concentrated in a 30 kDa molecular weight cutoff (MWCO) Amicon concentrator (Sigma Millipore), and then loaded onto a 24 ml Superdex 200 10/300 GL (GE Healthcare; catalog no.: 17-5174-01) size-exclusion column (SEC) equilibrated in 20 mM Tris (pH 8.0) (20 mM Hepes [pH 7.5] for FL SHIP1), 150 mM NaCl, 10% glycerol, 1 mM TCEP, and 0.05% CHAPS buffer. Peak fractions were concentrated in a 50 kDa MWCO Vivaspin 6 centrifuge tube and flash frozen at a final concentration of 1 to 30 μM using liquid nitrogen. Purification schemes for FL SHIP1, SHIP1(ΔSH2), and SHIP1(ΔCT) were identical. Biophysical parameters of FL SHIP1: pI = 7.37, ε_280_ = 106,690 M^−1^ cm^−1^, 133.3 kDa.

#### mNG-SHIP1(PH-PP-C2)

BL21(DE3) Star bacteria were transformed with his10-TEV-mNG-GGGGG-SHIP1 (mNG-PH-PP-C2; 191–878 aa) and plated on LB agar containing 50 μg/ml kanamycin. The following day, 50 ml of TPM media (20 g tryptone, 15 g yeast extract, 8 g NaCl, 2 g Na_2_HPO_4_, and 1 g KH_2_PO_4_ per liter) containing 50 μg/ml kanamycin was inoculated with one bacterial colony from the agar plate. This culture was grown for 12 to 16 h at 27 °C to an absorbance of 2 to 3 at 600 nm, before being diluted to an absorbance of 0.05 at 600 nm in 2 l of TPM media. These cultures were grown at 37 °C to an absorbance of 0.8 at 600 nm, shifted to 30 °C for 1 h, and then bacteria were induced with 50 μM IPTG to express mNG-SHIP1(PH-PP-C2). After 6 to 8 h of expression at 30 °C, bacterial cultures were harvested by centrifugation, transferred to 50 ml tubes by washing with 1× PBS (pH 7.2), pelleted, and then stored in the −80 °C freezer. For purification, frozen cell pellets were thawed in an ambient water bath and lysed into buffer containing 50 mM Na_2_HPO_4_ (pH 8.0), 400 mM NaCl, 1 mM PMSF, 0.4 mM BME, and 100 μg/ml DNase by microtip sonication (12–24 × 5 s pulses, 40% amplitude). Lysate was then centrifuged at 15,000 rpm (35,000*g*) for 60 min in a Beckman JA-20 rotor at 4 °C. During lysate centrifugation, a 5 ml HiTrap chelating column (Cat#) was charged with 50 ml of 100 mM CoCl_2_, generously washed with water (phosphate buffer will precipitate unchelated CoCl_2_ if not thoroughly removed), and equilibrated with lysis buffer lacking PMSF and DNase (>0.4 mM BME will destroy charged HiTrap column and turn column brown or black). HSS was then circulated over charged 5 ml HiTrap column for 2 h. Captured protein was washed with 100 ml of 50 mM Na_2_HPO_4_ (pH 8.0), 400 mM NaCl, 10 mM imidazole, and 0.4 mM BME and then eluted into buffer containing 500 mM imidazole. Peak fractions containing SHIP1 were pooled with 400 μl of 243 μM his6-TEV protease (S291V) with poly-R tail and dialyzed in 4 l of 25 mM Na_2_HPO_4_ (pH 8.0), 400 mM NaCl, and 0.4 mM BME buffer at 4 °C for ∼12 to 16 h to remove imidazole. The next day, the charged HiTrap column was equilibrated with dialysis buffer, and dialyzed protein was recirculated for 2 h at 4 °C to remove his6-TEV protease and uncleaved his10-TEV-mNG-GGGGG-SHIP1. Unbound protein was then concentrated in a 30 kDa MWCO Amicon concentrator (Sigma Millipore) and then loaded onto a 24 ml Superdex 200 10/300 GL (GE Healthcare; catalog no.: 17-5174-01) SEC equilibrated in 20 mM Tris (pH 8), 200 mM NaCl, 10% glycerol, and 1 mM TCEP buffer. Peak fractions were concentrated in a 30 kDa MWCO Vivaspin 6 centrifuge tube and flash frozen at a final concentration of 10 to 30 μM using liquid nitrogen. Biophysical parameters of mNG-SHIP1(PH-PP-C2): pI = 7.43, ε_280_ = 116,660 M^−1^ cm^−1^, 94.4 kDa.

#### Grp1 and TAPP1

The Grp1 PH domain derived from the human *CYTH3* gene was expressed in BL21(DE3) bacteria as a his6-MBP-N10-TEV-GGGG-Grp1(261–387 aa) fusion. When cloning this construct, a single cysteine was incorporated at the C terminus of the open reading frame for maleimide labeling. The TAPP1 PH domain derived from the human *PKHA1* gene was expressed in bacteria as either his6-MBP-TEV-GGGG-TAPP1 (182–303 aa) or his6-MBP-TEV-GGGG-TAPP1 (182–303 aa)-GGG-SNAP fusion. Protein expression was achieved by growing BL21(DE3) bacteria at 37 °C in 4 l of Terrific broth until an absorbance reached 0.8 at 600 nm. The cultures were then shifted to 18 °C for 1 h before inducing bacteria with 0.1 mM IPTG. Bacteria were grown for 20 h postinduction at 18 °C before harvesting cultures. For purification, bacteria were lysed by sonication into buffer containing 50 mM Na_2_HPO_4_ (pH 8.0), 400 mM NaCl, 0.4 mM BME, 1 mM PMSF, and 50 μg/ml DNase. Lysate was clarified by centrifugation at 16,000 rpm (35,000*g*) for 60 min in a Beckman JA-20 rotor at 4 °C. Soluble protein in clarified supernatant was circulated over a 5 ml HiTrap chelating column (GE Healthcare; catalog no.: 17-0409-01) that was preincubated with 100 mM CoCl_2_ for 10 min, washed with Milli-Q water, and equilibrated into lysis buffer lacking PMSF and DNase. After cell lysate circulated over the HiTrap column for 2 h, the column was washed with 100 ml of buffer containing 50 mM Na_2_HPO_4_ (pH 8.0), 300 mM NaCl, and 0.4 mM BME. Protein was eluted with buffer containing 50 mM Na_2_HPO_4_ (pH 8.0), 300 mM NaCl, and 500 mM imidazole at a flow rate of 4 ml/min. Peak fraction of HiTrap elution was combined with 100 μg/ml final concentration of TEV protease and dialyzed overnight against 4 l of buffer containing 20 mM Tris (pH 8.0), 200 mM NaCl, and 0.4 mM BME. The next day, we recirculated cleaved proteins over two HiTrap (Co^+2^) columns (2 × 5 ml) that were equilibrated in 50 mM Na_2_HPO_4_ (pH 8.0), 300 mM NaCl, and 0.4 mM BME containing buffer for 1 h. We concentrated the proteins *via* 10 kDa MWCO Vivaspin 20 to a volume of 5 ml. Concentrated proteins were then loaded and resolved on a Superdex 75 column equilibrated in 20 mM Tris (pH 8), 200 mM NaCl, 10% glycerol, and 1 mM TCEP. Peak fractions were pooled and concentrated to 375 μM (Grp1), 123 μM (TAPP1), and 32 μM (TAPP1-SNAP). All proteins were aliquoted and flash frozen with liquid nitrogen before storage in a −80 °C freezer. Biophysical parameters of GGGG-Grp1: pI = 8.00, ε_280_ = 30,940 M^−1^ cm^−1^, 15.5 kDa. Biophysical parameters of GGGG-TAPP1: pI = 8.91, ε_280_ = 16,960 M^−1^ cm^−1^, 14.4 kDa. Biophysical parameters of GGGG-TAPP1-GGG-SNAP: pI = 7.28, ε_280_ = 37,930 M^−1^ cm^−1^, 34.7 kDa.

#### LactC2, PLC*δ*, and SHIP1(PH-PP-C2)

As previously described, the coding sequence of human PLCδ-PH domain (11–140 aa) was expressed in BL21(DE3) Star bacteria as a his_6_-SUMO3-GGGG-PLCδ (11–140 aa) fusion protein (46, 47). The PS lipid sensor, LactC2, was expressed as a his6-TEV-SUMO3-GGGG-LactC2 (271–427 aa) fusion protein. When cloning LactC2 for recombinant protein expression, we incorporated a single cysteine at the C-terminal position of the LactC2 open reading frame. The central domain of SHIP1 was expressed as a his6-TEV-SUMO3-GGGG-SHIP1(PH-PP-C2, 292–878 aa) fusion. For all constructs, bacteria were grown at 37 °C in Terrific broth to an absorbance of 0.8 at 600 nm. Cultures were then shifted to 18 °C for 1 h, induced with 0.1 mM IPTG, and allowed to continue expressing protein for 20 h at 18 °C before being harvested. Frozen cell pellets containing recombinantly expressed proteins were individually thawed and lysed by sonication into buffer containing 50 mM Na_2_HPO_4_ (pH 8.0), 300 mM NaCl, 0.4 mM BME, 1 mM PMSF, and 100 μg/ml DNase. Lysate was then clarified by centrifugation at 16,000 rpm (35,172*g*) for 60 min in a Beckman JA-17 rotor chilled to 4 °C. Clarified lysates were circulated over 5 ml HiTrap Cobalt Chelating column (GE Healthcare; catalog no.: 17-0409-01) for 1 h at 4 °C. Bound proteins were eluted by applying a linear gradient of imidazole (0–500 mM, 8 CV, 40 ml total, 2 ml/min flow rate). Peak fractions were pooled, combined with 50 μg/ml SUMO protease (*i.e.*, his6-SenP2), and dialyzed against 4 l of buffer containing 50 mM Na_2_HPO_4_ (pH 8.0), 300 mM NaCl, and 0.4 mM BME for 16 to 18 h at 4 °C. The next day, each SUMO-cleaved protein was recirculated for 1 h over a 5 ml HiTrap Cobalt Chelating column to capture his6-SenP2 and his6-SUMO. Flow-through containing either GGGG-PLC***δ***, GGGG-LactC2, or GGGG-SHIP1(PH-PP-C2) was concentrated in a 10 kDa MWCO Vivaspin 20 and loaded on a Superdex 75 SEC equilibrated in 20 mM Tris (pH 8.0), 200 mM NaCl, 10% glycerol, and 1 mM TCEP. Peak fractions were pooled after SEC and concentrated to 375 μM (PLC***δ***), 123 μM (LactC2), and 32 μM (SHIP1, PH-PP-C2). All proteins were aliquoted and flash frozen with liquid nitrogen before storage in a −80 °C freezer. Biophysical parameters of GGGG-PLC***δ***: pI = 7.19, ε_280_ = 17,990 M^−1^ cm^−1^, 15.8 kDa. Biophysical parameters of GGGG-LactC2: pI = 8.99, ε_280_ = 44,920 M^−1^ cm^−1^, 18.1 kDa. Biophysical parameters of GGGG-SHIP1(PH-PP-C2): pI = 7.29, ε_280_ = 72,310 M^−1^ cm^−1^, 67.4 kDa.

#### YopH

BL21(DE3) were transformed with pCDF vector containing YopH (1–468 aa) lacking an affinity tag. We expressed and purified YopH as previously described (34). Bacteria were selected on LB agar plates containing 50 μg/ml streptomycin. For protein expression, bacteria were grown in Terrific broth at 37 °C to an absorbance of 0.8 at 600 nm and induced to express protein by adding 0.5 mM IPTG. Following 4 h of expression at 37 °C, bacteria were harvested by centrifugation. After decanting media, bacterial cell pellets were stored in the −80 °C freezer. To purify YopH, bacteria were lysed by microtip sonication into buffer containing 100 mM acetate (pH 5.7), 100 mM NaCl, 1 mM EDTA, 1 mM PMSF, 5 mM BME, and 50 μg/ml DNase. Lysate was clarified by centrifugation at 15,000 rpm (35,000*g*) for 60 min in a Beckman JA-20 rotor at 4 °C. Clarified lysate was then circulated over a 5 ml CM Sephadex (weak cation exchange) column for 1 h at 4 °C. The column was subsequently washed with 50 ml of buffer containing 100 mM acetate (pH 5.7), 100 mM NaCl, 1 mM EDTA, and 1 mM DTT. To elute YopH, we applied a linear gradient (100–500 mM NaCl) in the presence of 100 mM acetate (pH 5.7), 1 mM EDTA, and 1 mM DTT over a total volume of 100 ml at a flow rate of 2 ml/min. Peak fractions containing YopH were pooled, concentrated, and loaded on a Superdex 75 size-exclusion chromatography column equilibrated in buffer containing 20 mM Tris (pH 8.0), 100 mM NaCl, 5% glycerol, and 1 mM TCEP. Peak fractions containing YopH were pooled, concentrated, aliquoted, and flash frozen in liquid nitrogen. Biophysical parameters of YopH: pI = 8.92, ε_280_ = 18,910 M^−1^ cm^−1^, 50.9 kDa.

#### Src kinase

To obtain soluble recombinant chicken c-Src-family kinase, his_6_-TEV-Src (251–533 aa) was coexpressed with YopH (1–468 aa) in BL21(DE3) bacteria as previously described (45). Cotransformed bacteria were selected on LB agar plates containing 50 μg/ml streptomycin and 50 μg/ml kanamycin. For protein expression, bacteria were grown in Terrific broth at 37 °C to an absorbance of 1 at 600 nm. Bacterial cultures were then shifted to growth at 18 °C for 1 h before induction with 0.5 mM IPTG. Bacteria continued to express protein at 18 °C for 16 h before being harvested by centrifugation. Bacterial cell pellets were subsequently stored in −80 °C until initiating the purification. To purify his_6_-TEV-Src (251–533 aa), thawed bacterial pellets were lysed by sonication into buffer containing 50 mM Na_2_HPO_4_ (pH 8.0), 500 mM NaCl, 5% glycerol, 1 mM PMSF, 0.4 mM BME, and 50 μg/ml DNase. Cell lysate was clarified by centrifugation at 15,000 rpm (35,000*g*) for 60 min in a Beckman JA-20 rotor at 4 °C. The clarified lysate was then circulated over a 5 ml HiTrap Co^2+^ column using a peristaltic pump at 4 °C. Unbound protein was washed from the column with 100 ml of buffer containing 50 mM Na_2_HPO_4_ (pH 8.0), 500 mM NaCl, 5% glycerol, and 0.4 mM BME. Buffer containing 50 mM Na_2_HPO_4_ (pH 8.0), 500 mM NaCl, 5% glycerol, and 0.4 mM BME was used to elute his_6_-TEV-Src (251–533 aa). Eluate was combined with TEV protease and dialyzed against 4 l of 50 mM Na_2_HPO_4_ (pH 8.0), 400 mM NaCl, 5% glycerol, 0.4 mM BME at 4 °C for 16 h. Post TEV cleavage was recirculated back over the 5 ml HiTrap Co^2+^ column equilibrated in dialysis buffer. Next, Src (251–533 aa) was desalted into 20 mM Tris (pH 8.0), 50 mM NaCl, 5% glycerol, and 1 mM DTT using a G25 Sephadex column and then loaded on a 1 ml MonoQ anion exchange column. Src (251–533 aa) was eluted from the MonoQ with a linear gradient of NaCl (50–500 mM) over 50 ml at a flow rate of 1 ml/min. Peak fractions from the MonoQ were pooled, concentrated, and loaded on a Superdex 75 size-exclusion chromatography column equilibrated in 20 mM Tris (pH 8.0), 100 mM NaCl, 5% glycerol, and 1 mM TCEP. Fractions containing monomeric Src (251–533 aa) were pooled and concentrated to 94 μM, flash frozen in liquid nitrogen, and stored in the −80 °C freezer. Biophysical parameters of Src (251–533 aa): pI = 5.59, ε_280_ = 52,370 M^−1^ cm^−1^, 32.7 kDa.

### *In vitro* tyrosine phosphorylation and dephosphorylation

Activity of the promiscuous tyrosine phosphatase YopH was assessed *in vitro* by monitoring the dephosphorylation of membrane-conjugated ITIM peptides using TIRF microscopy. For these experiments, 20 nM of Cy3-SH2 domain was allowed to equilibrate with SLBs with maleimide lipid-conjugated ITIM peptide. The addition of 10 nM YopH rapidly dephosphorylated the ITIM peptide as indicated by the dissociation of the membrane-bound Cy3-SH2 (Fig. S4, *A*–*C*). The extent of tyrosine phosphorylation of insect cell–purified SHIP1 was assessed by Western blot following incubation with either YopH or c-Src kinase. To drive tyrosine phosphorylation, we combined 1 μM SHIP1 and 1 μM c-Src kinase. Dephosphorylation was achieved by combining 1 μM SHIP1 and 0.5 μM YopH. SHIP1 tyrosine phosphorylation and dephosphorylation reaction were performed in buffer containing 20 mM Hepes (pH 7.0), 150 mM NaCl, 1 mM ATP, 2 mM MgCl_2_, and 1 mM TCEP at 23 °C for 60 min. Reactions were quenched by adding 4× Laemmli buffer, followed by heat denaturation at 80 °C for 10 min. Proteins were resolved on a 4 to 12% Mini-PROTEAN TGX precast gradient gel (Bio-Rad; catalog no.: 4561094) at 200 V for 30 min. To visualize total protein, the resolved SDS-PAGE gel containing SHIP1, YopH, and c-Src was stained overnight in 1× SYPRO Ruby protein stain (ThermoFisher; catalog no.: S12000) following the manufacturer’s suggested protocol. The stained gel was visualized using Amersham Typhoon scanner (excitation = 450 nm, emission = 610 nm). Data are shown in Fig. S4*D*.

### Western blot

Purified SHIP1, YopH, and c-Src were resolved on a 4 to 12% Mini-PROTEAN TGX precast gradient gel (Bio-Rad; catalog no.: 4561094) at 200 V for 30 min. Proteins resolved by SDS-PAGE were transferred to nitrocellulose membranes (Bio-Rad; catalog no.: 1704271) using cold 1× transfer buffer (40 ml 5× transfer buffer [Bio-Rad; catalog no.: 1704271], 40 ml ethanol, and 120 ml Milli-Q water). Protein transfer to nitrocellulose membrane was accomplished using the 7 min mixed molecular weight protocol on the Bio-Rad Trans-Blot Turbo Transfer System (Bio-Rad; catalog no.: 1704150). Blots were washed with Milli-Q water and blocked for 1 h with intercept blocking buffer (LI-COR; catalog no.: 927-70001) at 23 °C. The nitrocellulose membrane was then incubated with 1:250 Alexa Fluor 546–labeled phosphotyrosine primary antibody (PY20) (Santa Cruz Biotechnology; catalog no.: sc-508-AF546) diluted in intercept blocking buffer and rocked overnight at 4 °C in the dark. Before visualization, the nitrocellulose membrane was washed four times for 5 min with 1× PBS plus 0.1% Tween-20. The Western blot was visualized using an Amersham Typhoon gel scanner. Data are shown in Fig. S4*D*.

### Fluorescent labeling of purified proteins

The following purified proteins were labeled *in vitro* using sortase-mediated peptide ligation: GGGG-PLC***δ***, GGGG-SHIP1(PH-PP-C2), and GGGG-TAPP1 as previously described (47, 48). In brief, an LPETGG peptide was labeled on the N-terminal amine with *N*-hydroxysuccinimide (NHS) fluorescent dye derivatives (*e.g.*, NHS-AF488) by combining 10 mM LPETGG peptide, 15 mM NHS-AF488 (or other fluorescent derivatives), and 30 mM triethylamine (Sigma; catalog no.: 471283) in anhydrous dimethyl sulfoxide (Sigma; catalog no.: 276855). Prior to labeling purified proteins, unreacted NHS fluorescent dyes were quenched with 50 mM Tris (pH 8.0). Protein labeling was achieved by combining 50 mM Tris (pH 8.0), 150 mM NaCl, 20 μM GGGG-protein, 100 μM AF488-LPETGG, and 10 μM his_6_-Sortase. This reaction mixture was incubated at 18 °C for 16 to 20 h, before removing free dye and peptide with G25 Sephadex desalting column. The final labeled proteins were separated from remaining fluorescent dye and labeled peptide using a Superdex 75 size-exclusion chromatography column equilibrated with 20 mM Tris (pH 8.0), 200 mM NaCl, 10% glycerol, and 1 mM TCEP. Peak fractions were pooled, concentrated to ∼20 μM, flash frozen in liquid nitrogen, and stored in the −80 °C freezer. We calculated the final concentration of AF488-GGGG-SHIP1(PH-PP-C2) using an adjusted A_280_ (A_280(protein)_ = A_280(measured)_ − A_494(AF488)_ ∗ 0.11) and the following extinction coefficients: ε_280(SHIP1)_ = 72,310 M^−1^•cm^−1^, ε_494(AF488)_ = 71,000 M^−1^•cm^−1^. We calculated the final concentration of Cy3-GGGG-TAPP1 using an adjusted A_280_ (A_280(protein)_ = A_280(measured)_ − A_550(Cy3)_ ∗ 0.086) and the following extinction coefficients: ε_280(TAPP1)_ = 16,960 M^−1^•cm^−1^, ε_550(Cy3)_ = 150,000 M^−1^•cm^−1^.

To fluorescently label TAPP1-SNAP, we combined 1 ml of 20 μM protein with 25 μM AF647-SNAP surface dye (New England Biolabs; catalog no.: S9136S). SNAP dye labeling was performed overnight at 4 °C in buffer containing 20 mM Tris (pH 8), 200 mM NaCl, 10% glycerol, and 1 mM TCEP. Labeled protein was partially separated from unreacted dye using a 5 kDa MWCO Amicon spin concentrator. The final labeled TAPP1-SNAP protein separated was resolved on a Superdex 75 size-exclusion chromatography column equilibrated with 20 mM Tris (pH 8.0), 200 mM NaCl, 10% glycerol, and 1 mM TCEP. Peak fractions were pooled, concentrated to ∼20 μM, flash frozen in liquid nitrogen, and stored in the −80 °C freezer. We calculate the final concentration of TAPP1-SNAP-AF647 using an adjusted A_280_ (*i.e.*, A_280(protein)_ = A_280(observed)_ – A_650(dye)_ ∗ 0.109) and the following extinction coefficients: ε_280(TAPP1-SNAP)_ = 37,930 M^−1^•cm^−1^, ε_650(AF647)_ = 237,000 M^−1^•cm^−1^.

LactC2 and Grp1 were labeled *in vitro* on a single C-terminal cysteine residue using Dyomics 647 maleimide (Dyomics GmbH; catalog no.: 647P1-01) or Alexa Fluor 647 maleimide (ThermoFisher; catalog no.: A20347), respectively. Labeling reactions were performed by combining 20 μM of protein with 100 μM maleimide dye on ice for 60 min. Labeling reactions were quenched by adding 10 mM DTT for 15 min. Excess unreacted dye was removed from labeled protein using a PD-10 G25 Sephadex desalting column. The final labeled proteins were separated from unreacted fluorescent dye on a Superdex 75 size-exclusion chromatography column equilibrated with 20 mM Tris (pH 8.0), 200 mM NaCl, 10% glycerol, and 1 mM TCEP.

### Preparation of small unilamellar vesicles

The following lipids were used to generate small unilamellar vesicles (SUVs): 1,2-dioleoyl-*sn*-glycero-3-phosphocholine (18:1 DOPC; Avanti, catalog no.: 850375C), 1,2-dioleoyl-*sn*-glycero-3-phospho-l-serine (18:1; Avanti, catalog no.: 840035C), 1,2-dioleoyl-*sn*-glycero-3-phosphoethanolamine (Avanti, catalog no.: 850725C), 1,2-dioleoyl-*sn*-glycero-3-phosphoethanolamine-*N*-[4-(p-maleimidomethyl)cyclohexane-carboxamide] (18:1 MCC-PE; Avanti, catalog no,: 780201C), d-myo-phosphatidylinositol 3,4,5-trisphosphate (PI(3,4,5)P_3_ diC16; Echelon, catalog no.: P-3916-100ug). Lipids from Avanti were purchased as single-use ampules containing between 0.1 and 5 mg of lipids dissolved in chloroform. PI(3,4,5)P_3_ lipids from Echelon were purchased as salts and solubilized in the manufacturer’s suggested solvent containing 263 μl CHCl_3_, 526 μl MeOH, and 211 μl H_2_O per ml volume. To make liposomes, 2 μmol total lipids are combined in a 35 ml glass round bottom flask containing 2 ml of chloroform. Lipids are dried to a thin film using rotary evaporation with the glass round-bottom flask submerged in a 42 °C water bath. After evaporating all the chloroform, the round bottom flask was placed under a vacuum for 15 min. The lipid film was then resuspended in 2 ml of PBS (pH 7.2), making a final concentration of 1 mM total lipids. All lipid mixtures expressed as percentages (*e.g.*, 98% DOPC, 2% PI(3,4,5)P_3_) are equivalent to molar fractions. For example, a 1 mM lipid mixture containing 98% DOPC and 2% PI(3,4,5)P_3_ is equivalent to 0.98 mM DOPC and 0.02 mM PI(3,4,5)P_3_. To generate 30 to 50 nm SUVs, 1 mM total lipid mixtures were extruded through a 0.05 μm pore size 19 mm polycarbonate (PC) membrane (Sigma; catalog no.: WHA800308) with filter supports (Avanti; catalog no.: 610014) on both sides of the PC membrane. Hydrated lipids at a concentration of 1 mM were extruded through the PC membrane 11 times.

### Preparation of SLBs

SLBs are formed on 25 × 75 mm coverglass (IBIDI; catalog no.: 10812) as previously described (46). In brief, coverglass was first cleaned with 2% Hellmanex III (ThermoFisher; catalog no.: 14-385-864) and then etched with Pirahna solution (1:3, hydrogen peroxide:sulfuric acid) for 5 to 10 min. Etched coverglass, washed extensively with Milli-Q water again, is rapidly dried with nitrogen gas before adhering to a 6-well sticky-side chamber (IBIDI; catalog no.: 80608). SLBs were formed by flowing 0.25 mM total lipid concentration of 50 nm SUVs diluted in 1× PBS (pH 7.2) into an assembled IBIDI chamber. SUVs were incubated in the IBIDI chamber for 30 min and then washed with 4 ml of PBS (pH 7.4) to remove nonabsorbed SUVs. Membrane defects are blocked for 10 min with a solution of 1 mg/ml beta casein (ThermoFisher; catalog no.: 37528) diluted in 1× PBS (pH 7.2). After blocking, membranes were washed with 1× PBS and stored for up to 3 h in 1× PBS before flowing in reaction buffer and imaging.

### Conjugation of pY peptide to supported membranes

SLBs with MCC-PE lipids were used to covalently couple phosphorylated peptides to the maleimide lipid headgroups. Phosphorylated peptides (**C**KTEAENTIT(pY)SLIK; Elim Biopharmaceuticals) correspond to the ITIM sequence derived from FcγRIIB (19). A cysteine (bold) was added to the N terminus of the peptide to form a covalent interaction with the maleimide lipid headgroup of the MCC-PE lipids. We refer to this peptide as pY-ITIM throughout the article. For these SLBs, 100 μl of 10 μM pY-ITIM peptides diluted in a 1× PBS (pH 7.2) and 0.1 mM TCEP buffer was added to the IBIDI chamber and incubated for 2 h. The addition of 0.1 mM TCEP significantly increased the coupling efficiency of pY-ITIM. SLBs with MCC-PE lipids were then washed with 2 ml of 5 mM BME diluted in 1× PBS (pH 7.4) and incubated for 15 min to destroy the unreacted maleimide headgroups. SLBs were washed with 1 ml of 1× PBS, followed by 1 ml of kinase buffer before TIRF-M.

### Oxygen scavenging system

Glucose oxidase (32 mg/ml, 100× stock) and catalase (5 mg/ml, 100× stock) were solubilized in 20 mM Hepes (pH 7.0), 150 mM NaCl, 10% glycerol, and 1 mM TCEP buffer, and then flash frozen in liquid nitrogen and stored at −80 °C. Trolox (200 mM, 100× stock) is UV treated and stored at −20 °C. Approximately 10 min before imaging, 100× oxygen scavenger stocks were diluted in kinase buffer containing enzymes/biosensors to achieve a final concentration of 320 μg/ml glucose oxidase (Serva; catalog no.: 22780.01, *Aspergillus niger*), 50 μg/ml catalase (Sigma; catalog no.: C40-100MG, bovine liver), and 2 mM Trolox (Cayman Chemicals; catalog no.: 10011659). Trolox was prepared using a previously described protocol that utilized UV irradiation to drive the formation of a quinone species (47, 49).

### Kinetics measurements of PI(3,4)P_2_ production

The kinetics of PI(3,4,5)P_3_ dephosphorylation *via* SHIP1 was measured on SLBs formed in IBIDI chambers and visualized using TIRF microscopy. The reaction buffer contains 20 mM Hepes (pH 7.0), 150 mM NaCl, 1 mM ATP, 5 mM MgCl_2_, 0.5 mM EGTA, 200 μg/ml beta casein, 20 mM BME, and 20 mM glucose. Most kinetics assays used initial membrane compositions of 96% DOPC, 2% PI(3,4,5)P_3_, and 2% MCC-PE, or 86% DOPC, 10% 1,2-dioleoyl-*sn*-glycero-3-phospho-l-serine, 2% PI(3,4,5)P_3_, and 2% MCC-PE. Membrane compositions that varied from the aforementioned conditions are described in figure legends. As indicated in the figure legend, we measured the conversion of PI(3,4,5)P_3_ to PI(3,4)P_2_ by visualizing the membrane localization of either 20 nM Grp1-AF647 (PH domain; PI(3,4,5)P_3_ sensor) or 6 nM Cy3-TAPP1 (PH domain; PI(3,4)P_2_ sensor) with TIRF-M. By convention, enzyme kinetics are plotted in terms of product formation rather than substrate depletion. For experiments that utilize the Grp1 sensor, normalized kinetic traces were inverted to show the production of PI(3,4)P_2_ lipids. Assuming a footprint of 0.72 nm^2^ as reported for DOPC lipids (50), we calculated a density of 27,778 lipids/μm^2^ for 2% PI(3,4)P_2_. Apparent rate constants (*k*_app_) were calculated using the half-time relationship for first-order reactions: *t*_1/2_ = 0.693/*k*_app_.

### Cell culture

PLB-985 neutrophil-like cells are an established model cell line for human neutrophils and were obtained from the laboratory of Dr Sean Collins (University of California at Davis). PLB-985 cells were grown in suspension in RPMI1640 + GlutaMAX media containing 25 mM Hepes (Life Technologies; catalog no.: 72400047), 9% fetal bovine serum (FBS), penicillin (100 units/ml) (Life Technologies; catalog no.: 15140122), and streptomycin (100 μg/ml) (Life Technologies; catalog no.: 15140122). Cell lines were grown in humidified incubators at 37 °C in the presence of 5% CO_2_ and split three times per week, keeping densities between 0.1 and 2 × 10^6^ cells/ml. PLB-985 cells were differentiated into a neutrophil-like state by culturing 0.2 × 10^6^ cells/ml for 6 to 7 days in RPMI media supplemented with 2% FBS, penicillin (100 units/ml), streptomycin (100 μg/ml), 1.3% dimethyl sulfoxide, and 2% Nutridoma-CS (Sigma; catalog no.: 11363743001). Nutridoma-CS was added to increase the chemotactic response of the cells, but this supplement is optional (51).

Human embryonic kidney (HEK) 293T Lenti-X were obtained from Takara (catalog no.: 632180). These cells transformed with adenovirus type 5 DNA and expresses the SV40 large T antigen. HEK293T Lenti-X cells were cultured in Dulbecco’s modified Eagle’s medium (DMEM) + GlutMAX + high glucose (4.5 g/l) + sodium pyruvate (110 mg/l) (Life Technologies; catalog no.: 10569010) supplemented with 10% FBS (Sigma; catalog no.: F4135-500ML), penicillin (100 units/ml), and streptomycin (100 μg/ml). Cells were grown in 10 cm dishes in humidified incubators at 37 °C in the presence of 5% CO_2_ and split at a confluency of 80 to 90% every 2 to 3 days. HEK293T Lenti-X cells were split using 1.5 ml of 0.25% trypsin. Trypsin was that quenched with 8.5 ml complete DMEM containing 10% FBS. Cells were diluted 1:10 and seeded on a new 10 cm dish containing a total volume of 10 ml complete DMEM warmed to 37 °C.

### Lentivirus production

Lentivirus was generated by transfecting 60 to 70% confluent HEK293 Lenti-X cells in a 10 cm plate containing 8 ml of complete media. Transfection reagents were prepared by combining 6.7 μg psPAX2 (second-generation lentiviral packaging plasmi; Addgene #12260), 0.85 μg pVSV-G (expresses VSV-G envelop protein for pseudotyping NanoMEDIC particle; Addgene #138479) (44), 7.5 μg of transfer lentiviral vector containing gene of interest, and 30 μl of 1 mg/ml polyethyleneimine) in 0.5 ml Opti-Mem (ThermoFisher; catalog no.: 31985070). This mixture was incubated for 15 min at room temperature before adding dropwise to plated HEK293 Lenti-X cells. Media containing lentivirus were harvested 48 h after initiating transfection and clarified by centrifugation to remove cell debris. Lentivirus was concentrated by adding 333 μl Lenti-X concentrator (Takara; catalog no.: 631231) per ml of viral supernatant, incubated overnight at 4 °C, and then centrifuged at 1500*g* for 45 min. Supernatant was aspirated, and the white viral-containing pellet was resuspended in 0.4 ml of complete RPMI media (9% FBS). Lentivirus was used immediately or stored at −80 °C. To infect PLB-985 cells, 0.4 ml of concentrated lentivirus was added with 5 ml of undifferentiated cells at a density of 0.2 × 10^6^ cells/ml containing a final concentration of 8 μg/ml polybrene (Millipore; catalog no.: TR-1003-G, 10 mg/ml stock, 1250×) in the cell culture media. The cells were passaged at least one time before differentiating into the neutrophil-like state (see *Cell culture* section for protocol).

### Live cell imaging

Differentiated PLB-985 cells were prepared for imaging by centrifuging 0.5 ml of cells at 100*g* for 10 min, aspirating off the medium, and resuspending the cells in warm 1x HBSS imaging media (20 mM HEPES [pH 7.2], 150 mM NaCl, 4 mM KCl, 1 mM MgCl_2_, 10 mM glucose, 0.2% bovine serum albumin). Differentiated PLB-985 cells were then imaged using fibronectin-coated glass attached to an IBIDI flow cell chamber (52). To image cells, 25 × 75 mm coverslips were cleaned with 2% Hellmanex III (ThermoFisher; catalog no.: 14-385-864), washed with Milli-Q water, dried with N_2_ gas, and attached to an IBIDI flow cell chamber (IBIDI sticky-Slide VI 0.4; catalog no.: 80608). A 10 μg/ml fibronectin (Sigma; catalog no.: F1141, 1 mg/ml stock concentration) solution diluted in 1× PBS was added to each well of the IBIDI chamber and incubated for 60 min at 23 °C. Unbound fibronectin was washed out with 1× PBS and then blocked with 0.2% bovine serum albumin dissolved in 1× PBS (fatty/endotoxin free; Sigma; catalog no.: B4501) for 15 min. Differentiated PLB-985 cells centrifuged and suspended in imaging media (aforementioned recipe) were flowed into the IBIDI chamber and allowed to adhere for 10 to 15 min. Cells were visualized by TIRF-M in imaging media and stimulated with a final uniform concentration of 10 nM fMLF (Sigma, catalog no.: F3506) to drive PI(3,4,5)P_3_ production.

### Microscope hardware and imaging acquisition

Membrane binding and lipid dephosphorylation reactions reconstituted on SLBs were visualized using an inverted Nikon Eclipse Ti2 microscope using a 100× Nikon (1.49 numerical aperture) oil immersion TIRF objective. TIRF-M images of SLBs were acquired using an iXion Life 897 EMCCD camera (Andor Technology Ltd). Fluorescently labeled proteins were excited with either a 488 nm, 561 nm, or 637 nm diode laser (OBIS laser diode; Coherent, Inc) controlled with a Vortran laser drive with acousto-optic tunable filters control. The power output measured through the objective for single-particle imaging was 1 to 2 mW. Excitation light was passed through the following dichroic filter cubes before illuminating the sample: (1) ZT488/647rpc and (2) ZT561rdc (ET575LP) (Semrock). Fluorescence emission was detected on the iXion Life 897 EMCCD camera positioned after a Nikon emission filter wheel housing the following emission filters: ET525/50M, ET600/50M, and ET700/75M (Semrock). All experiments were performed at room temperature (23 °C) for both biochemistry and live-cell imaging. Microscope hardware was controlled by Nikon NIS elements.

### Single-particle tracking algorithm

Single-particle detection and tracking was performed using the TrackMate plugin (53) on ImageJ/Fiji as previously described (46, 47). Output files are analyzed using Prism 9 graphing software (GraphPad by Dotmatics). To calculate the single-molecule dwell times for fluorescently labeled SHIP1 and various PIP lipid-binding domain, we generated a cumulative distribution frequency plot using the frame interval as the bin size (*e.g.*, 12–50 ms). The log_10_(1-cumulative distribution frequency) was plotted against the dwell time and fit to either a single or double exponential decay curve.

Single exponential model:

$$f\left(t\right)=e^{\left(-x/\tau \right)}$$

Two exponential model:

$$f\left(t\right)={\alpha *e}^{\left(-x/\tau 1\right)}{+\left(1-\alpha \right)*e}^{\left(-x/\tau 2\right)}$$

Fitting procedure initiated with a single exponential. In cases of a low-quality single exponential fit, a maximum of two populations was used. For double exponential fit, alpha (α) represents the percentage of short dwelling molecules characterized by the time constant, ***τ***_1_.

## Data availability

All the information needed for interpretation of the data is presented in the article or the supporting information. Plasmids related to this work are available upon request.

## Supporting information

This article contains supporting information (45, 47).

## Conflict of interest

The authors declare that they have no conflicts of interest with the contents of this article.
