# Supplementary material for: Mechanisms controlling membrane recruitment and activation of the autoinhibited SHIP1 inositol 5-phosphatase
Source: J Biol Chem. 2023 Jul 7;299(8):105022. doi: 10.1016/j.jbc.2023.105022 (PMC10448276; doi:10.1016/j.jbc.2023.105022)
Supplement: Supporting information [file mmc10.pdf]

SUPPLEMENTAL INFORMATION

Mechanisms controlling membrane recruitment and activation of the autoinhibited SHIP1 inositol 5-phosphatase

Grace L. Waddell[1,2], Emma E. Drew[1,2], Henry P. Rupp[1,2], and Scott D. Hansen[1,2*]

[1] Department of Chemistry and Biochemistry, University of Oregon, Eugene, OR 97403
[2] Institute of Molecular Biology, University of Oregon, Eugene, OR 97403

*Corresponding author:
shansen5@uoregon.edu

Supplemental Figure 1

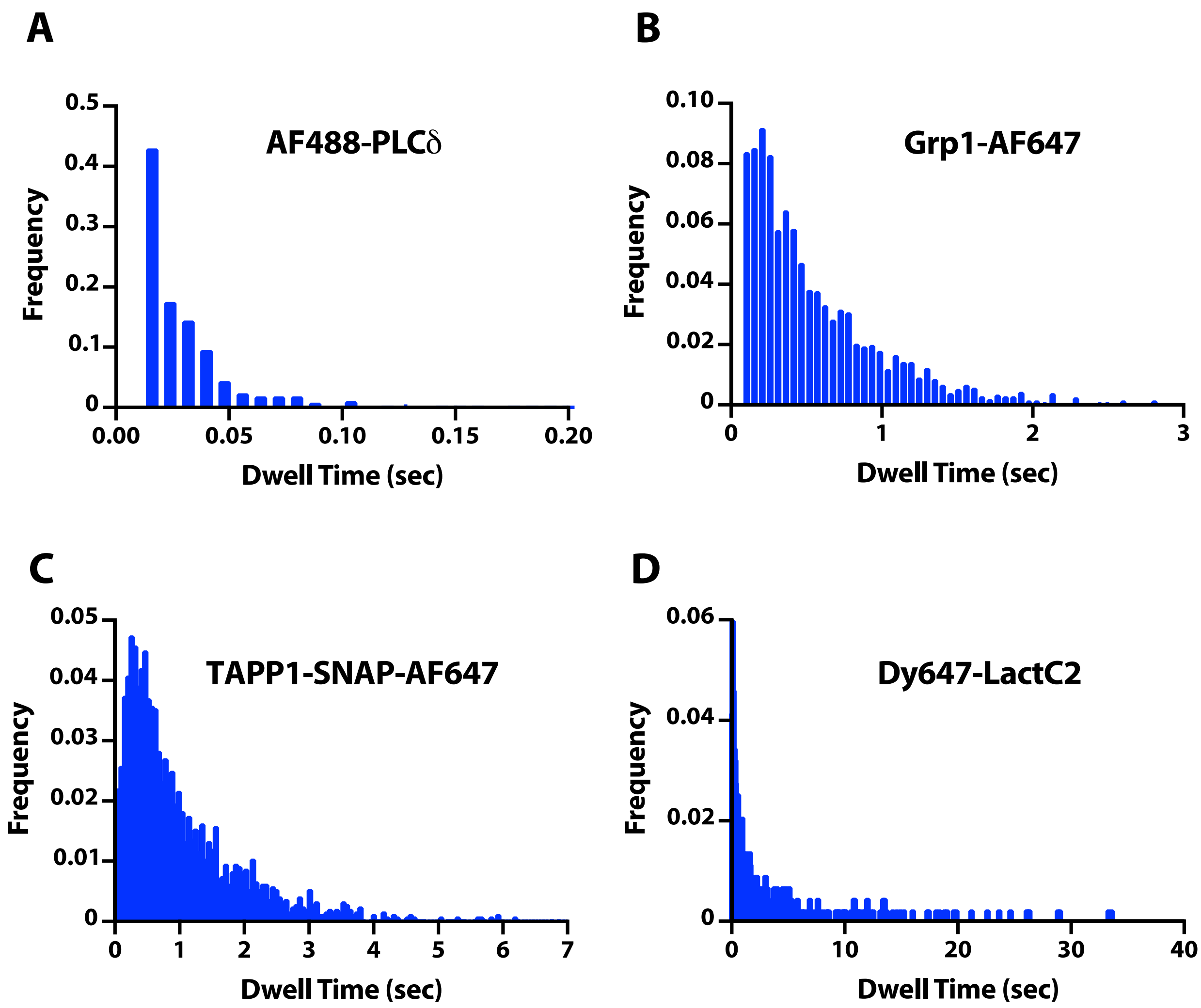

Supplementary Figure 1

Single molecule dwell time distributions of PIP lipid binding domains measured on supported membranes using smTIRF-M

(A-D) Representative dwell time frequency distributions for data shown in Figure 1E. Data was collected under the following conditions: **(A)** 250 pM AF488-PLCδ + 2% PI(4,5)P_2, **(B)** 1 pM Grp1-AF647 + 2% PI(3,4,5)P_3, **(C)** 1 pM TAPP1-SNAP-AF647 + 2% PI(3,4)P_2, **(D)** 2 pM LactC2-Dy647 + 20% DOPS. This data is presented in Figure 1E yielding the following dwell times: AF488-PLCδ (τ_1 = 24 ± 2 ms) TAPP1-SNAP-AF647 (τ_1 = 1.02 ± 0.053 s), Grp1-AF647 (τ_1 = 0.544 ± 0.007 s), or LactC2-Dy647 (τ_1 = 0.765 ± 0.191 s, τ_2 = 6.58 ± 0.539 s, α = 0.5 ± 0.04). Note that the exponential curve fits are not shown. Membrane composition: DOPC lipids plus the indicated PIP lipids concentration stated above.

Supplemental Figure 2

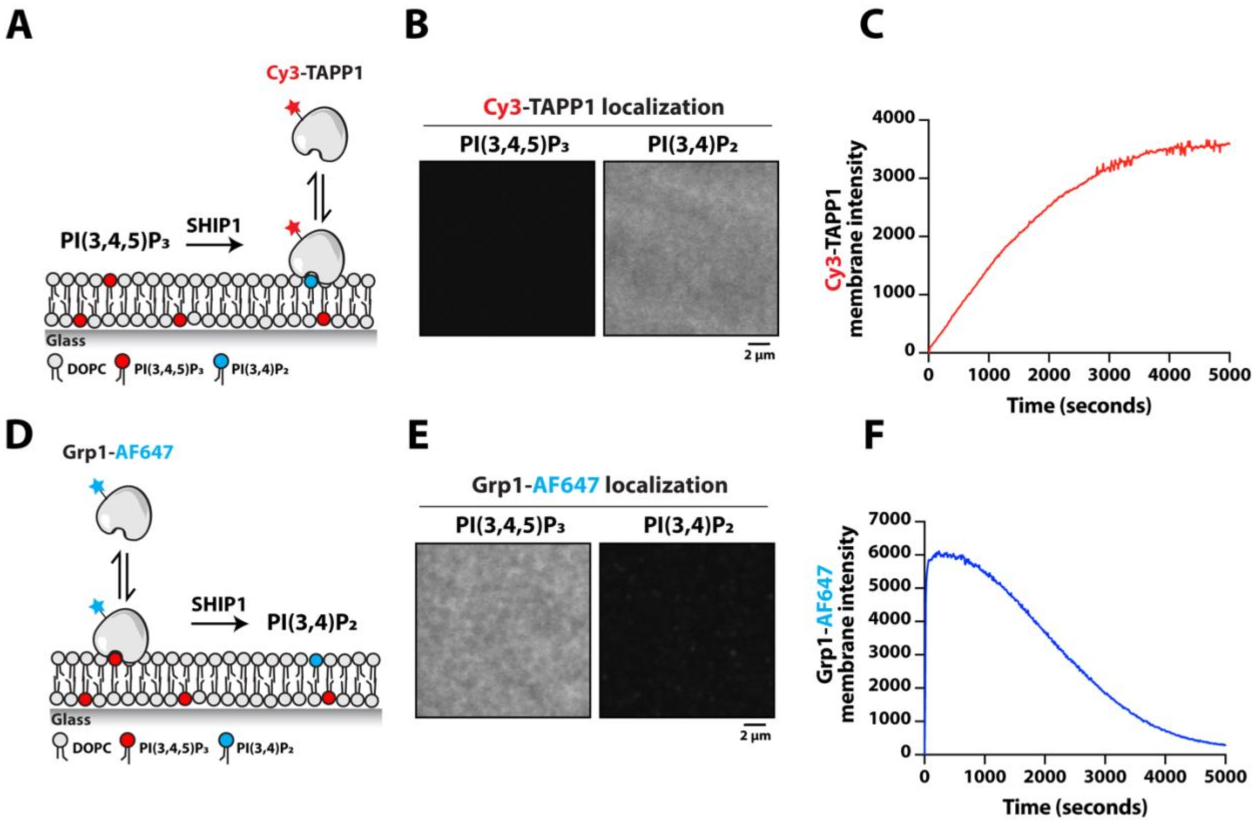

Supplementary Figure 2

Validation of TAPP1 and Grp1 sensors for measuring dephosphorylation of PI(3,4,5)P_3

(A) Cartoon schematic showing membrane association of Cy3-TAPP1 in a PI(3,4)P_2 dependent manner. **(B)** Representative TIRF-M images showing the localization of 20 nM Cy3-TAPP1 on SLBs containing 98% DOPC with either 2% PI(3,4,5)P_3 or 2% PI(3,4)P_2. **(C)** Kinetics of PI(3,4,5)P_3 dephosphorylation measured in the presence of 50 nM full-length SHIP1 and 50 nM Cy3-TAPP1. **(D)** Cartoon schematic showing membrane association of Grp1-AF647 in a PI(3,4,5)P_3 dependent manner. **(E)** Representative TIRF-M images showing the localization of 20 nM Grp1-AF647 on SLBs containing 98% DOPC with either 2% PI(3,4,5)P_3 or 2% PI(3,4)P_2. **(F)** Kinetics of PI(3,4,5)P_3 dephosphorylation measured in the presence of 50 nM full-length SHIP1 and 20 nM Grp1-AF647.

Supplemental Figure 3

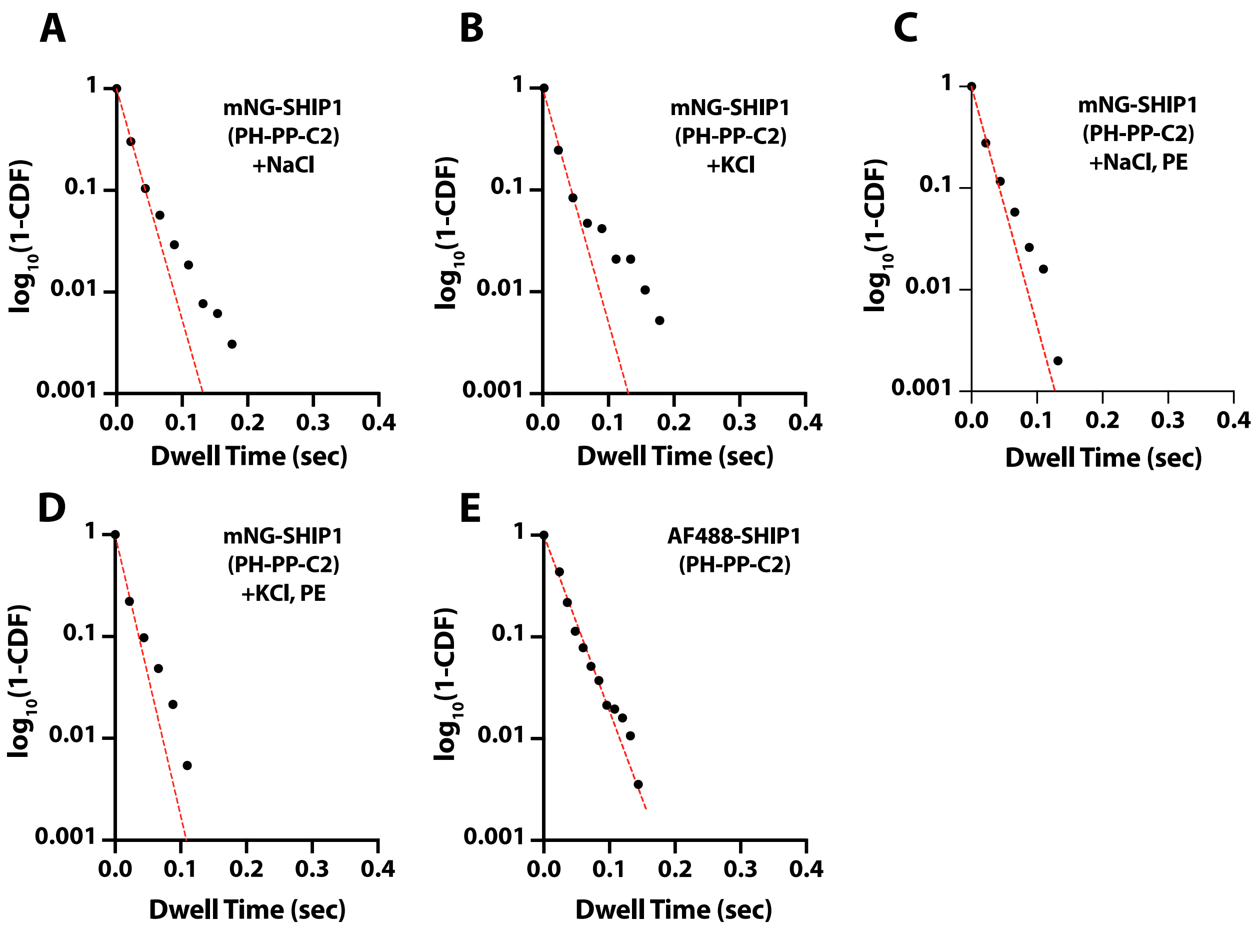

Supplementary Figure 3

Single molecule membrane binding dynamics of SHIP1(PH-PP-C2)

(A-D) Single molecule dwell time distributions measured in the presence of 500 pM mNG-SHIP1(PH-PP-C2) on supported lipid bilayers in the presence of either **(A-B)** NaCl or **(C-D)** KCl buffer. “NaCl” buffer = 150 mM NaCl, plus components in reaction buffer (*see Methods*). “KCl” buffer = 140 mM KCl, 10 mM NaCl, plus components in reaction buffer (*see Methods*). Data plotted as dwell time versus $\log_{10}$(1-cumulative distribution frequency). Curves are fit with a single exponential decay curves yielding the following dwell times for mNG-SHIP1(PH-PP-C2): **(A)** τ_1 = 20 ± 1 ms (n = 2069), **(B)** τ_1 = 24 ± 1 ms (n = 642), **(C)** τ_1 = 18 ± 1 ms (n = 1503), **(D)** τ_1 = 17 ± 2 ms (n = 528), **(E)** τ_1 = 24 ± 1 ms (n = 6167). Dwell times are reported as mean ± SD from N = 3-4 technical replicates. **(A-B, E)** Membrane composition: 78% DOPC, 20% DOPS, 2% PI(3,4,5)P_3. **(C-D)** Membrane composition: 39% DOPC, 39% DOPE, 20% DOPS, 2% PI(3,4,5)P_3.

Supplemental Figure 4

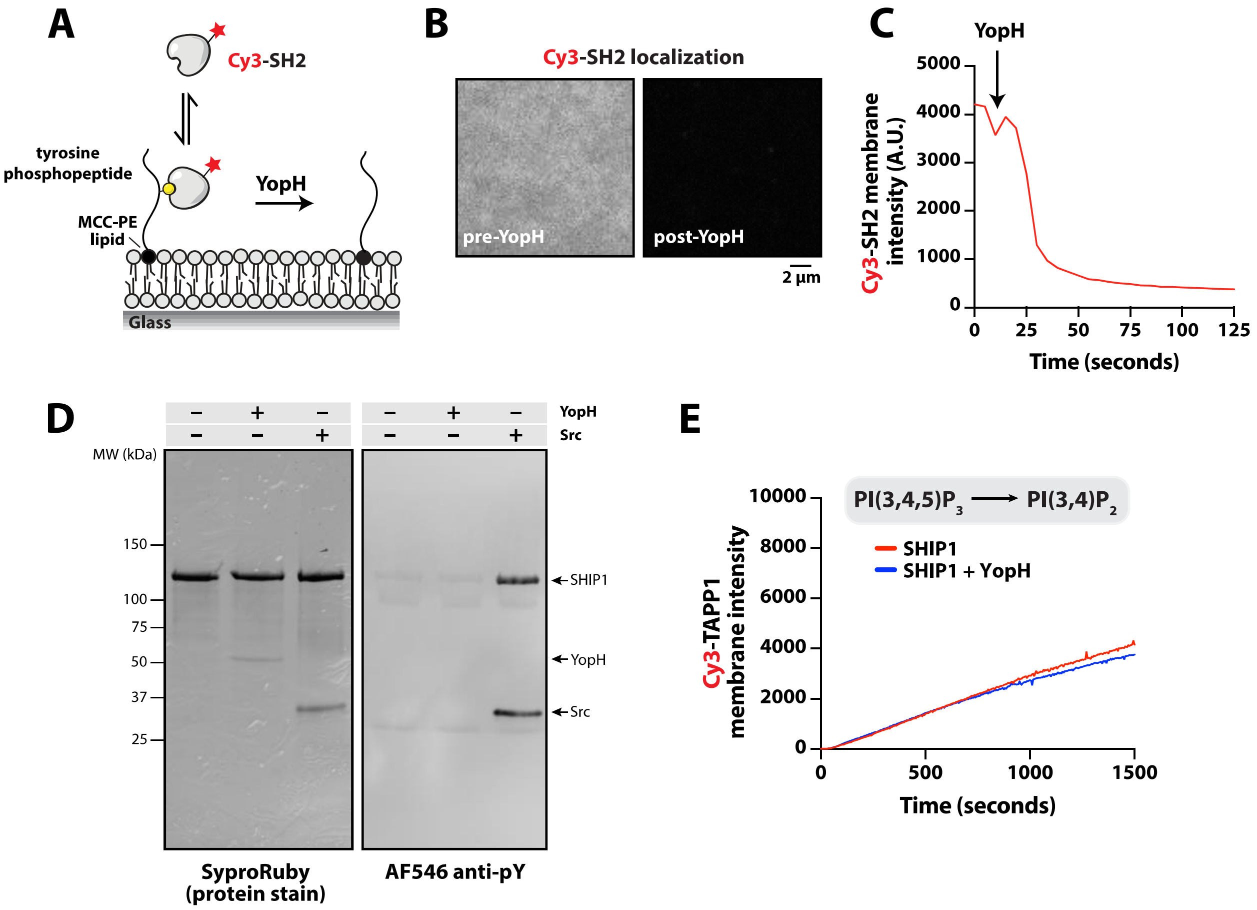

Supplementary Figure 4

Tyrosine phosphatase, YopH, does not enhance activity of full length SHIP1

(A) Cartoon schematic showing membrane association of Cy3-SH2 with membrane anchored phosphotyrosine peptide derived from a ITIM motif (pY-ITIM). The tyrosine phosphatase, YopH, drives the dissociation of Cy3-SH2 following dephosphorylation of the pY-ITIM peptide. **(B)** Representative TIRF-M images showing the localization of 50 nM Cy3-SH2 in the absence and presence of 10 nM YopH. **(C)** Kinetics of phosphotyrosine (pY-ITIM) peptide dephosphorylation monitored in the presence of 50 nM Cy3-SH2 and 10 nM YopH. **(D)** Full length SHIP1 (1-1188aa) purified from insect cells is not tyrosine phosphorylated but it can be phosphorylated in vitro with purified c-Src kinase. The SDS-PAGE gel was loaded with 10 µL of samples containing different combinations of 1 µM SHIP1(1-1188aa), 0.5 nM YopH, and 1 µM Src kinase. Total protein was detected by staining SDS-PAGE gel with SyproRuby stain. Tyrosine phosphorylation was detected by Western Blot using a AF546 labeled anti-pY antibody. **(E)** Activity of full-length SHIP1 is not modulated by the addition of the tyrosine phosphatase YopH. Reactions contained 50 nM SHIP1 (FL), 20 nM Cy3-TAPP1, 50 nM YopH was incubated for 10 minutes before injecting in SLB chamber and imaged using TIRF-M. **(B-C)** Membrane composition: 96% DOPC, 2% PI(3,4,5)P_3, 2% MCC-PE-(pY conjugated). **(E)** Membrane composition: 98% DOPC, 2% PI(3,4,5)P_3.

TABLE S1

Protein visualized	Membrane composition	τ_1 ± SD (sec)	τ_2 ± SD (sec)	α ± SD	*N*	*n*
AF488-PLCδ	2% PI(4,5)P_2	0.024 ± 0.002	—	—	4	389
Grp1-AF647	2% PI(3,4,5)P_3	0.544 ± 0.005	—	—	3	5588
TAPP1-SNAP-AF647	2% PI(3,4)P_2	1.02 ± 0.053	—	—	3	8102
LactC2-Dy647	20% PS	0.765 ± 0.191	6.58 ± 0.539	0.50 ± 0.04	3	1167
mEos-LactC2	PM	0.371 ± 0.069	—	—	12	18124
mEos-Grp1	PM	0.392 ± 0.048	—	—	10	10184
mEos-SHIP1 (PH-PP-C2)	PM	0.038 ± 0.003	—	—	4	8480
mEos-SHIP1 (PH-PP-C2)	PM + fMLF	0.037 ± 0.005	—	—	5	7975
AF488-SHIP1 (PH-PP-C2)	2% PI(3,4,5)P_3, 20% PS	0.024 ± 0.001			4	6167
mNG-SHIP1 (PH-PP-C2)	2% PI(3,4,5)P_3, 20% PS	0.025 ± 0.001	—	—	2	1562
mNG-SHIP1 (PH-PP-C2)	2% PI(3,4,5)P_3, 20% PS*	0.009 ± 0.002	0.056 ± 0.007	0.44 ± 0.13	5	4801
mNG-SHIP1 (FL)	2% PI(3,4,5)P_3, pY(mb)	0.054 ± 0.004	0.872 ± 0.082	0.62 ± 0.02	3	3702
mNG-SHIP1 (ΔCT)	2% PI(3,4,5)P_3, pY(mb)	0.055 ± 0.005	0.941 ± 0.017	0.44 ± 0.13	2	2046

SD = standard deviation from the indicated number of technical replicates
N = # of SLBs or cells used for calculating the mean dwell times (i.e. technical replicates)
n = total number of molecules tracked across the indicated number of technical replicates (*N*)
alpha (α) = fraction of molecules with characteristic dwell time (τ_1 and τ_2)
membrane composition: DOPC plus the indicated PIP/PS lipid concentrations.
Membrane composition for live cell imaging is called PM (plasma membrane).
Cells were stimulated with 10 nM chemoattractant (fMLF).
* = assay buffer contained 75 mM NaCl instead of 150 mM NaCl.

SUPPLEMENTAL MOVIE LEGENDS

Supplemental Movie 1
Supported membrane binding dynamics of AF488-PLCδ PH domain visualized by smTIRF microscopy. Movie is associated with data in Figure 1D-1E. Video plays at 20 frames per second. Data was collected with 8 ms time intervals (i.e. 125 fps). Scale bar is 2 µm. Membrane composition: 98% DOPC, 2% PI(4,5)P_2.

Supplemental Movie 2
Supported membrane binding dynamics of TAPP1-SNAP-AF647 PH domain visualized by smTIRF microscopy. Movie is associated with data in Figure 1D-1E. Video plays at 20 frames per second. Data was collected with 50 ms time intervals (i.e. 20 fps). Scale bar is 2 µm. Membrane composition: 98% DOPC, 2% PI(3,4)P_2.

Supplemental Movie 3
Supported membrane binding dynamics of Grp1-AF647 PH domain visualized by smTIRF microscopy. Movie is associated with data in Figure 1D-1E. Video plays at 20 frames per second. Data was collected with 50 ms time intervals (i.e. 20 fps). Scale bar is 2 µm. Membrane composition: 98% DOPC, 2% PI(3,4,5)P_3.

Supplemental Movie 4
Supported membrane binding dynamics of LactC2-Dy647 visualized by smTIRF microscopy. Movie is associated with data in Figure 1D-1E. Video plays at 20 frames per second. Data was collected with 50 ms time intervals (i.e. 20 fps). Scale bar is 2 µm. Membrane composition: 90% DOPC, 10% PS.

Supplemental Movie 5
Plasma membrane binding dynamics of mEos3.2-Grp1, a PI(3,4,5)P_3 lipid sensor, visualized by smTIRF microscopy in differentiated PLB-985 neutrophil-like cells. Movie is associated with data in Figure 2E. Video plays at 20 frames per second. Data was collected with 52 ms time intervals (i.e. 19 fps). Scale bar is 2 µm.

Supplemental Movie 6
Plasma membrane binding dynamics of mEos3.2-LactC2, a PS lipid sensor, visualized by smTIRF microscopy in differentiated PLB-985 neutrophil-like cells. Movie is associated with data in Figure 2E. Video plays at 20 frames per second. Data was collected with 52 ms time intervals (i.e. 19 fps). Scale bar is 2 µm.

Supplemental Movie 7
Plasma membrane binding dynamics of mEos3.2-SHIP1(PH-PP-C2) visualized by smTIRF microscopy in differentiated PLB-985 neutrophil-like cells. Cells were imaged in the absence (left) or presence of 10 nM fMLF chemoattractant (right). Movie is associated with data in Figure 2H-2I. Video plays at 20 frames per second. Data was collected with 22 ms time intervals (i.e. 45 fps). Scale bar is 2 µm.

Supplemental Movie 8
Supported membrane binding dynamics of mNG-SHIP1(PH-PP-C2) visualized by smTIRF microscopy. Movie is associated with data in Figure 3G. Video plays at 20 frames per second. Data was collected with 12 ms time intervals (i.e. 83 fps). Scale bar is 2 µm. Membrane composition: 78% DOPC, 2% PI(3,4,5)P_3, 20% PS.

Supplemental Movie 9
Supported membrane binding dynamics of mNG-SHIP1(1-118aa) bound to conjugated pY peptide visualized by smTIRF microscopy. Movie is associated with data in Figure 5E. Video plays at 10 frames per second. Data was collected with 35 ms time intervals. Scale bar is 2 µm. Membrane composition: 96% DOPC, 2% PI(3,4,5)P_3, 2% MCC-PE/pY.

PLASMID INVENTORY

Recombinant DNA			
his6-TEV-SUMO3-GGGGG-PLCδ PH domain (11-140aa)	bacterial	Hansen et al. 2019	pSH450
his6-MBP-N10-TEV-GGGGG-Grp1 (261-387aa)	bacterial	This paper	pSH558
his6-MBP-TEV-GGGG-TAPP1 (182-303aa)	bacterial	This paper	pSH1012
his6-MBP-TEV-GGGG-TAPP1 (182-303aa)-GGG-SNAP	bacterial	This paper	pSH1258
his6-TEV-SUMO3-GGGGG-LactC2 (271-427aa)	bacterial	This paper	pSH1271
his10-**mNG**-GGGGG-SHIP1 (PH-PP-C2, 292-878aa)	bacterial	This paper	pSH1082
his6-TEV-SUMO3-GGGG-SHIP1 (PH-PP-C2, 292-878aa)	bacterial	This paper	pSH1340
his6-TEV-SHIP1 FL (1-1188aa)	baculovirus	This paper	pSH798
his6-TEV-**mNG**-SHIP1 FL (1-1188aa)	baculovirus	This paper	pSH973
his6-TEV-**mNG**-SHIP1 ΔCT (1-878aa)	baculovirus	This paper	pSH1042
his6-TEV-**mNG**-SHIP1 ΔSH2 (102-1188aa)	baculovirus	This paper	pSH1053
psPAX2 (2nd generation lentiviral packaging vector)	lentivirus	Addgene, 12260	pSH1224
pVSV-G (VSV-G envelop protein)	lentivirus	Addgene, 138479	pSH1226
Ubc-**mEos3.2**-(GGGGS)x2-Grp1 (261-387aa)	lentivirus	This paper	pSH1252
Ubc-**mEos3.2**-(GGGGS)x2-LactC2 (271-427aa)	lentivirus	This paper	pSH1267
Ubc-**mEos3.2**-(GGGGS)x2-SHIP1 (PH-PP-C2, 292-878aa)	lentivirus	This paper	pSH1254
his6-TEV-c-Src (251-533aa)	bacterial	Seeliger et al. 2005	pSH594
YopH (1-468aa)	bacterial	Seeliger et al. 2005	pSH595

Hansen SD, Huang WYC, Lee YK, Bieling P, Christensen SM, Groves JT. 2019. Stochastic geometry sensing and polarization in a lipid kinase–phosphatase competitive reaction. *Proc Natl Acad Sci USA* **116**:15013–15022. doi:10.1073/pnas.1901744116

Seeliger MA, Young M, Henderson MN, Pellicena P, King DS, Falick AM, Kuriyan J. 2005. High yield bacterial expression of active c-Abl and c-Src tyrosine kinases. *Protein Science.* **14**:3135–3139. doi:10.1110/ps.051750905

PEPTIDE SEQUENCES

The protein sequences of recombinantly expressed and purified proteins used in the study are shown below. The following tags were cleaved off the recombinant proteins and removed during the purification:

his6-TEV-SUMO3 (↓= site of SUMO protease cleavage)
MKHHHHHHPMSDYDIPTTENLYFQGAMGNDHINLKVAGQDGSVVQFKIKRHTPLSKLMKAYCE
RQGLSMRQIRFRFDGQPINETDTPAQLEMEDEDTIDVFQQQTGG↓GGGG

his6-MBP-N10-TEV (↓= site of TEV protease cleavage)
MGSSHHHHHHGSSMKIEEGKLVIWINGDKGYNGLAEVGKKFEKDTGIKVTVEHPDKLEEKFPQ
VAATGDGPDIIFWAHDRFGGYAQSGLLAEITPDKAFQDKLYPFTWDAVRYNGKLIAYPIAVEALS
LIYNKDLLPNPPKTWEEIPALDKELKAKGKSALMFNLQEPYFTWPLIAADGGYAFKYENGKYDIK
DVGVDNAGAKAGLTFLVDLIKNKHMNADTDYSIAEAAFNKGETAMTINGPWAWSNIDTSKVNY
GVTVLPTFKGQPSKPFVGVLSAGINAASPNKELAKEFLENYLLTDEGLEAVNKDKPLGAVALKS
YEEELAKDPRIAATMENAQKGEIMPNIPQMSAFWYAVRTAVINAASGRQTVDEALKDAQTNSS
SNNNNNNNNNNLGIEENLYFQ↓GGGGG

his6-TEV (↓= site of TEV protease cleavage)
MGSSHHHHHHENLYFQ↓SN

his10-TEV (↓= site of TEV protease cleavage)
MKHHHHHHHHHHYDIPTTENLYFQ↓GA

Shown below are the plasmid #'s and names of the full-length recombinant proteins that were expressed and purified in this study. The affinity and solubility tags that are underlined in the gene names were cleaved off the indicated protein and removed through purification. The primary amino acid sequence shown below represents the final purified product.

pSH450, his6-TEV-SUMO3-GGGG-PLCδ PH domain (11-140aa)
…GGGGTSHGLQDDEDLQALLKGSQLLKVKSSSWRRERFYKLQEDCKTIWQESRKVMRTPES
QLFSIEDIQEVRMGHRTEGLEKFARDVPEDRCFSIVFKDQRNTLDLIAPSPADAQHWVLGLHKII
HHSGSMDQRQKGS*

pSH558, his6-MBP-N10-TEV-GGGG-Grp1 PH domain (261-387aa) Cys light mutant
…GGGGGTSTFFNPDREGWLLKLGGRVKTWKRRWFILTDNSLYYFEYTTDKEPRGIIPLENLSIREVEDP
RKPNSFELYNPSHKGQVIKASKTEADGRVVEGNHVVYRISAPSPEEKEEWMKSIKASISRDPFYDMC*

pSH1271, his6-TEV-SUMO3-GGGG-LactC2 (271-427aa)
…GGGGTEPLGLKDNTIPNKQITASSYYKTWGLSAFSWFPYYARLDNQGKFNAWTAQTNSASE
WLQIDLGSQKRVTGIITQGARDFGHIQYVAAYRVAYGDDGVTWTEYKDPGASESKIFPGNMDN
NSHKKNIFETPFQARFVRIQPVAWHNRITLRVELLGC*

pSH1012, his6-MBP-N10-TEV-GGGG-TAPP1 (182-303aa)
…GGGGGTSFTPKPPQDSAVIKAGYCVKQGAVMKNWKRRYFQLDENTIGYFKSELEKEPLRVI
PLKEVHKVQECKQSDIMMRDNLFEIVTTSRTFYVQADSPEEMHSWIKAVSGAIVAQRGPGRSA
SSEHP*

pSH1258, his6-MBP-N10-TEV-GGGG-TAPP1 (182-303aa)-SNAP
…GGGGGTSFTPKPPQDSAVIKAGYCVKQGAVMKNWKRRYFQLDENTIGYFKSELEKEPLRVI
PLKEVHKVQECKQSDIMMRDNLFEIVTTSRTFYVQADSPEEMHSWIKAVSGAIVAQRGPGRSA
SSEHPTGGGMDKDCEMKRTTLDSPLGKLELSGCEQGLHEIKLLGKGTSAADAVEVPAPAAVLG
GPEPLMQATAWLNAYFHQPEAIEEFPVPALHHPVFQQESFTRQVLWKLLKVVKFGEVISYQQL
AALAGNPAATAAVKTALSGNPVPILIPCHRVVSSSGAVGGYEGGLAVKEWLLAHEGHRLGKPG
LGPAGIGAPGS*

pSH594, his6-TEV-Src (251-533aa)
…GHMQTQGLAKDAWEIPRESLRLEVKLGQGCFGEVWMGTWNGTTRVAIKTLKPGTMSPEAF
LQEAQVMKKLRHEKLVQLYAVVSEEPIYIVTEYMSKGSLLDFLKGEMGKYLRLPQLVDMAAQIA
SGMAYVERMNYVHRDLRAANILVGENLVCKVADFGLARLIEDNEYTARQGAKFPIKWTAPEAA
LYGRFTIKSDVWSFGILLTELTTKGRVPYPGMVNREVLDQVERGYRMPCPPECPESLHDLMCQ
CWRKDPEERPTFEYLQAFLEDYFTSTEPQYQPGENL*

pSH595, YopH (1-468aa)
MNLSLSDLHRQVSRLVQQESGDCTGKLRGNVAANKETTFQGLTIASGARESEKVFAQTVL
SHVANVVLTQEDTAKLLQSTVKHNLNNYDLRSVGNGNSVLVSLRSDQMTLQDAKVLLEAA
LRQESGARGHVSSHSHSALHAPGTPVREGLRSHLDPRTPPLPPRERPHTSGHHGAGEARA
TAPSTVSPYGPEARAELSSRLTTLRNTLAPATNDPRYLQACGGEKLNRFRDIQCCRQTAV
RADLNANYIQVGNTRTIACQYPLQSQLESHFRMLAENRTPVLAVLASSSEIANQRFGMPD
YFRQSGTYGSITVESKMTQQVGLGDGIMADMYTLTIREAGQKTISVPVVHVGNWPDQTAV
SSEVTKALASLVDQTAETKRNMYESKGSSAVGDDSKLRPVIHCRAGVGRTAQLIGAMCMN
DSRNSQLSVEDMVSQMRVQRNGIMVQKDEQLDVLIKLAEGQGRPLLNS*

pSH798, his6-TEV-SHIP1 (1-1188aa)
…SNVPCWNHGNITRSKAEELLSRTGKDGSFLVRASESISRAYALCVLYRNCVYTYRILPNEDD
KFTVQASEGVSMRFFTKLDQLIEFYKKENMGLVTHLQYPVPLEEEDTGDDPEEDTESVVSPPE
LPPRNIPLTASSCEAKEVPFSNENPRATETSRPSLSETLFQRLQSMDTSGLPEEHLKAIQDYLST
QLAQDSEFVKTGSSSLPHLKKLTTLLCKELYGEVIRTLPSLESLQRLFDQQLSPGLRPRPQVPG
EANPINMVSKLSQLTSLLSSIEDKVKALLHEGPESPHRPSLIPPVTFEVKAESLGIPQKMQLKVD
VESGKLIIKKSKDGSEDKFYSHKKILQLIKSQKFLNKLVILVETEKEKILRKEYVFADSKKREGFCQ
LLQQMKNKHSEQPEPDMITIFIGTWNMGNAPPPKKITSWFLSKGQGKTRDDSADYIPHDIYVIG
TQEDPLSEKEWLEILKHSLQEITSVTFKTVAIHTLWNIRIVVLAKPEHENRISHICTDNVKTGIANT
LGNKGAVGVSFMFNGTSLGFVNSHLTSGSEKKLRRNQNYMNILRFLALGDKKLSPFNITHRFTH
LFWFGDLNYRVDLPTWEAETIIQKIKQQQYADLLSHDQLLTERREQKVFLHFEEEEITFAPTYRF
ERLTRDKYAYTKQKATGMKYNLPSWCDRVLWKSYPLVHVVCQSYGSTSDIMTSDHSPVFATF
EAGVTSQFVSKNGPGTVDSQGQIEFLRCYATLKTKSQTKFYLEFHSSCLESFVKSQEGENEEG
SEGELVVKFGETLPKLKPIISDPEYLLDQHILISIKSSDSDESYGEGCIALRLEATETQLPIYTPLTH
HGELTGHFQGEIKLQTSQGKTREKLYDFVKTERDESSGPKTLKSLTSHDPMKQWEVTSRAPP
CSGSSITEIINPNYMGVGPFGPPMPLHVKQTLSPDQQPTAWSYDQPPKDSPLGPCRGESPPTP
PGQPPISPKKFLPSTANRGLPPRTQESRPSDLGKNAGDTLPQEDLPLTKPEMFENPLYGSLSS
FPKPAPRKDQESPKMPRKEPPPCPEPGILSPSIVLTKAQEADRGEGPGKQVPAPRLRSFTCSS
SAEGRAAGGDKSQGKPKTPVSSQAPVPAKRPIKPSRSEINQQTPPTPTPRPPLPVKSPAVLHL
QHSKGRDYRDNTELPHHGKHRPEEGPPGPLGRTAMQ*

pSH973, his6-TEV-mNeonGreen-(GGGGS)x2-SHIP1 FL (1-1188aa)
…SNTGMVSKGEEDNMASLPATHELHIFGSINGVDFDMVGQGTGNPNDGYEELNLKSTKGDLQ
FSPWILVPHIGYGFHQYLPYPDGMSPFQAAMVDGSGYQVHRTMQFEDGASLTVNYRYTYEGS
HIKGEAQVKGTGFPADGPVMTNSLTAADWCRSKKTYPNDKTIISTFKWSYTTGNGKRYRSTAR
TTYTFAKPMAANYLKNQPMYVFRKTELKHSKTELNFKEWQKAFTDVMGMDELYKGGGGSGG

GGSTSVPCWNHGNITRSKAEELLSRTGKDGSFLVRASESISRAYALCVLYRNCVYTYRILPNED
DKFTVQASEGVSMRFFTKLDQLIEFYKKENMGLVTHLQYPVPLEEEDTGDDPEEDTESVVSPP
ELPPRNIPLTASSCEAKEVPFSNENPRATETSRPSLSETLFQRLQSMDTSGLPEEHLKAIQDYLS
TQLAQDSEFVKTGSSSLPHLKKLTTLLCKELYGEVIRTLPSLESLQRLFDQQLSPGLRPRPQVP
GEANPINMVSKLSQLTSLLSSIEDKVKALLHEGPESPHRPSLIPPVTFEVKAESLGIPQKMQLKV
DVESGKLIIKKSKDGSEDKFYSHKKILQLIKSQKFLNKLVILVETEKEKILRKEYVFADSKKREGFC
QLLQQMKNKHSEQPEPDMITIFIGTWNMGNAPPPKKITSWFLSKGQGKTRDDSADYIPHDIYVI
GTQEDPLSEKEWLEILKHSLQEITSVTFKTVAIHTLWNIRIVVLAKPEHENRISHICTDNVKTGIAN
TLGNKGAVGVSFMFNGTSLGFVNSHLTSGSEKKLRRNQNYMNILRFLALGDKKLSPFNITHRFT
HLFWFGDLNYRVDLPTWEAETIIQKIKQQQYADLLSHDQLLTERREQKVFLHFEEEEITFAPTYR
FERLTRDKYAYTKQKATGMKYNLPSWCDRVLWKSYPLVHVVCQSYGSTSDIMTSDHSPVFAT
FEAGVTSQFVSKNGPGTVDSQGQIEFLRCYATLKTKSQTKFYLEFHSSCLESFVKSQEGENEE
GSEGELVVKFGETLPKLKPIISDPEYLLDQHILISIKSSDSDESYGEGCIALRLEATETQLPIYTPLT
HHGELTGHFQGEIKLQTSQGKTREKLYDFVKTERDESSGPKTLKSLTSHDPMKQWEVTSRAP
PCSGSSITEIINPNYMGVGPFGPPMPLHVKQTLSPDQQPTAWSYDQPPKDSPLGPCRGESPPT
PPGQPPISPKKFLPSTANRGLPPRTQESRPSDLGKNAGDTLPQEDLPLTKPEMFENPLYGSLS
SFPKPAPRKDQESPKMPRKEPPPCPEPGILSPSIVLTKAQEADRGEGPGKQVPAPRLRSFTCS
SSAEGRAAGGDKSQGKPKTPVSSQAPVPAKRPIKPSRSEINQQTPPTPTPRPPLPVKSPAVLH
LQHSKGRDYRDNTELPHHGKHRPEEGPPGPLGRTAMQ*

pSH1042, his6-TEV-mNeonGreen-(GGGGS)x2-SHIP1 (1-878aa)
…SNTGMVSKGEEDNMASLPATHELHIFGSINGVDFDMVGQGTGNPNDGYEELNLKSTKGDLQ
FSPWILVPHIGYGFHQYLPYPDGMSPFQAAMVDGSGYQVHRTMQFEDGASLTVNYRYTYEGS
HIKGEAQVKGTGFPADGPVMTNSLTAADWCRSKKTYPNDKTIISTFKWSYTTGNGKRYRSTAR
TTYTFAKPMAANYLKNQPMYVFRKTELKHSKTELNFKEWQKAFTDVMGMDELYKGGGGSGG
GGSTSVPCWNHGNITRSKAEELLSRTGKDGSFLVRASESISRAYALCVLYRNCVYTYRILPNED
DKFTVQASEGVSMRFFTKLDQLIEFYKKENMGLVTHLQYPVPLEEEDTGDDPEEDTESVVSPP
ELPPRNIPLTASSCEAKEVPFSNENPRATETSRPSLSETLFQRLQSMDTSGLPEEHLKAIQDYLS
TQLAQDSEFVKTGSSSLPHLKKLTTLLCKELYGEVIRTLPSLESLQRLFDQQLSPGLRPRPQVP
GEANPINMVSKLSQLTSLLSSIEDKVKALLHEGPESPHRPSLIPPVTFEVKAESLGIPQKMQLKV
DVESGKLIIKKSKDGSEDKFYSHKKILQLIKSQKFLNKLVILVETEKEKILRKEYVFADSKKREGFC
QLLQQMKNKHSEQPEPDMITIFIGTWNMGNAPPPKKITSWFLSKGQGKTRDDSADYIPHDIYVI
GTQEDPLSEKEWLEILKHSLQEITSVTFKTVAIHTLWNIRIVVLAKPEHENRISHICTDNVKTGIAN
TLGNKGAVGVSFMFNGTSLGFVNSHLTSGSEKKLRRNQNYMNILRFLALGDKKLSPFNITHRFT
HLFWFGDLNYRVDLPTWEAETIIQKIKQQQYADLLSHDQLLTERREQKVFLHFEEEEITFAPTYR
FERLTRDKYAYTKQKATGMKYNLPSWCDRVLWKSYPLVHVVCQSYGSTSDIMTSDHSPVFAT
FEAGVTSQFVSKNGPGTVDSQGQIEFLRCYATLKTKSQTKFYLEFHSSCLESFVKSQEGENEE
GSEGELVVKFGETLPKLKPIISDPEYLLDQHILISIKSSDSDESYGEGCIALRLEATETQLPIYTPLT
HHGELTGHFQGEIKLQTSQGKTREKLYDFVKTERDESSGPK*

pSH1053, his6-TEV-mNeonGreen-(GGGGS)x2-SHIP1 (ΔSH2, 102-1188aa)
…SNTGMVSKGEEDNMASLPATHELHIFGSINGVDFDMVGQGTGNPNDGYEELNLKSTKGDLQ
FSPWILVPHIGYGFHQYLPYPDGMSPFQAAMVDGSGYQVHRTMQFEDGASLTVNYRYTYEGS
HIKGEAQVKGTGFPADGPVMTNSLTAADWCRSKKTYPNDKTIISTFKWSYTTGNGKRYRSTAR
TTYTFAKPMAANYLKNQPMYVFRKTELKHSKTELNFKEWQKAFTDVMGMDELYKGGGGSGG
GGSTSPLEEEDTGDDPEEDTESVVSPPELPPRNIPLTASSCEAKEVPFSNENPRATETSRPSLS
ETLFQRLQSMDTSGLPEEHLKAIQDYLSTQLAQDSEFVKTGSSSLPHLKKLTTLLCKELYGEVIR
TLPSLESLQRLFDQQLSPGLRPRPQVPGEANPINMVSKLSQLTSLLSSIEDKVKALLHEGPESP
HRPSLIPPVTFEVKAESLGIPQKMQLKVDVESGKLIIKKSKDGSEDKFYSHKKILQLIKSQKFLNK
LVILVETEKEKILRKEYVFADSKKREGFCQLLQQMKNKHSEQPEPDMITIFIGTWNMGNAPPPK
KITSWFLSKGQGKTRDDSADYIPHDIYVIGTQEDPLSEKEWLEILKHSLQEITSVTFKTVAIHTLW

NIRIVVLAKPEHENRISHICTDNVKTGIANTLGNKGAVGVSFMFNGTSLGFVNSHLTSGSEKKLR
RNQNYMNILRFLALGDKKLSPFNITHRFTHLFWFGDLNYRVDLPTWEAETIIQKIKQQQYADLLS
HDQLLTERREQKVFLHFEEEEITFAPTYRFERLTRDKYAYTKQKATGMKYNLPSWCDRVLWKS
YPLVHVVCQSYGSTSDIMTSDHSPVFATFEAGVTSQFVSKNGPGTVDSQGQIEFLRCYATLKT
KSQTKFYLEFHSSCLESFVKSQEGENEEGSEGELVVKFGETLPKLKPIISDPEYLLDQHILISIKS
SDSDESYGEGCIALRLEATETQLPIYTPLTHHGELTGHFQGEIKLQTSQGKTREKLYDFVKTER
DESSGPKTLKSLTSHDPMKQWEVTSRAPPCSGSSITEIINPNYMGVGPFGPPMPLHVKQTLSP
DQQPTAWSYDQPPKDSPLGPCRGESPPTPPGQPPISPKKFLPSTANRGLPPRTQESRPSDLG
KNAGDTLPQEDLPLTKPEMFENPLYGSLSSFPKPAPRKDQESPKMPRKEPPPCPEPGILSPSIV
LTKAQEADRGEGPGKQVPAPRLRSFTCSSSAEGRAAGGDKSQGKPKTPVSSQAPVPAKRPIK
PSRSEINQQTPPTPTPRPPLPVKSPAVLHLQHSKGRDYRDNTELPHHGKHRPEEGPPGPLGRT
AMQ*

pSH1082, his10-TEV-mNeonGreen-(GGGGG)-SHIP1 PH-PP-C2 (292-878aa)
…GAMVSKGEEDNMASLPATHELHIFGSINGVDFDMVGQGTGNPNDGYEELNLKSTKGDLQFS
PWILVPHIGYGFHQYLPYPDGMSPFQAAMVDGSGYQVHRTMQFEDGASLTVNYRYTYEGSHI
KGEAQVKGTGFPADGPVMTNSLTAADWCRSKKTYPNDKTIISTFKWSYTTGNGKRYRSTARTT
YTFAKPMAANYLKNQPMYVFRKTELKHSKTELNFKEWQKAFTDVMGMDELYKGGGGGTSPSL
IPPVTFEVKAESLGIPQKMQLKVDVESGKLIIKKSKDGSEDKFYSHKKILQLIKSQKFLNKLVILVE
TEKEKILRKEYVFADSKKREGFCQLLQQMKNKHSEQPEPDMITIFIGTWNMGNAPPPKKITSWF
LSKGQGKTRDDSADYIPHDIYVIGTQEDPLSEKEWLEILKHSLQEITSVTFKTVAIHTLWNIRIVVL
AKPEHENRISHICTDNVKTGIANTLGNKGAVGVSFMFNGTSLGFVNSHLTSGSEKKLRRNQNY
MNILRFLALGDKKLSPFNITHRFTHLFWFGDLNYRVDLPTWEAETIIQKIKQQQYADLLSHDQLL
TERREQKVFLHFEEEEITFAPTYRFERLTRDKYAYTKQKATGMKYNLPSWCDRVLWKSYPLVH
VVCQSYGSTSDIMTSDHSPVFATFEAGVTSQFVSKNGPGTVDSQGQIEFLRCYATLKTKSQTK
FYLEFHSSCLESFVKSQEGENEEGSEGELVVKFGETLPKLKPIISDPEYLLDQHILISIKSSDSDE
SYGEGCIALRLEATETQLPIYTPLTHHGELTGHFQGEIKLQTSQGKTREKLYDFVKTERDESSG
PK*

pSH1340, his6-TEV-SUMO3-GGGG-SHIP1 (PH-PP-C2, 292-878aa)
…GGGGPSLIPPVTFEVKAESLGIPQKMQLKVDVESGKLIIKKSKDGSEDKFYSHKKILQLIKSQK
FLNKLVILVETEKEKILRKEYVFADSKKREGFCQLLQQMKNKHSEQPEPDMITIFIGTWNMGNAP
PPKKITSWFLSKGQGKTRDDSADYIPHDIYVIGTQEDPLSEKEWLEILKHSLQEITSVTFKTVAIH
TLWNIRIVVLAKPEHENRISHICTDNVKTGIANTLGNKGAVGVSFMFNGTSLGFVNSHLTSGSEK
KLRRNQNYMNILRFLALGDKKLSPFNITHRFTHLFWFGDLNYRVDLPTWEAETIIQKIKQQQYAD
LLSHDQLLTERREQKVFLHFEEEEITFAPTYRFERLTRDKYAYTKQKATGMKYNLPSWCDRVL
WKSYPLVHVVCQSYGSTSDIMTSDHSPVFATFEAGVTSQFVSKNGPGTVDSQGQIEFLRCYAT
LKTKSQTKFYLEFHSSCLESFVKSQEGENEEGSEGELVVKFGETLPKLKPIISDPEYLLDQHILISI
KSSDSDESYGEGCIALRLEATETQLPIYTPLTHHGELTGHFQGEIKLQTSQGKTREKLYDFVKTE
RDESSGPK*

Lentiviral constructs expressed in PLB-985 cells:

pSH1252, mEos3.2-(GGGGS)x2-Grp1 (261-387aa)
MSAIKPDMKIKLRMEGNVNGHHFVIDGDGTGKPFEGKQSMDLKVKEGGPLPFAFDILTTAFHY
GNRVFAKYPDNIQDYFKQSFPKGYSWERSLTFEDGGICNARNDITMEGDTFYNKVRFYGTNFP
ANGPVMQKKTLKWEPSTEKMYVRDGVLTGDIEMALLLEGNAHYRCDFRTTYKAKEKGVKLPG
AHFVDHCIEILSHDKDYNKVKLYEHAVAHSGLPDNARRGGGGSGGGGSTGTFFNPDREGWLL
KLGGRVKTWKRRWFILTDNCLYYFEYTTDKEPRGIIPLENLSIREVEDPRKPNCFELYNPSHKG
QVIKACKTEADGRVVEGNHVVYRISAPSPEEKEEWMKSIKASISRDPFYDM*

pSH1267, mEos3.2-(GGGGS)x2-LactC2 (271-427aa)
MSAIKPDMKIKLRMEGNVNGHHFVIDGDGTGKPFEGKQSMDLKVKEGGPLPFAFDILTTAFHY
GNRVFAKYPDNIQDYFKQSFPKGYSWERSLTFEDGGICNARNDITMEGDTFYNKVRFYGTNFP
ANGPVMQKKTLKWEPSTEKMYVRDGVLTGDIEMALLLEGNAHYRCDFRTTYKAKEKGVKLPG
AHFVDHCIEILSHDKDYNKVKLYEHAVAHSGLPDNARRGGGGSGGGGSTGCTEPLGLKDNTIP
NKQITASSYYKTWGLSAFSWFPYYARLDNQGKFNAWTAQTNSASEWLQIDLGSQKRVTGIITQ
GARDFGHIQYVAAYRVAYGDDGVTWTEYKDPGASESKIFPGNMDNNSHKKNIFETPFQARFV
RIQPVAWHNRITLRVELLGC*

pSH1254, mEos3.2-(GGGGGS)x2-SHIP1 PH-PP-C2 (292-878aa)
MSAIKPDMKIKLRMEGNVNGHHFVIDGDGTGKPFEGKQSMDLKVKEGGPLPFAFDILTTAFHY
GNRVFAKYPDNIQDYFKQSFPKGYSWERSLTFEDGGICNARNDITMEGDTFYNKVRFYGTNFP
ANGPVMQKKTLKWEPSTEKMYVRDGVLTGDIEMALLLEGNAHYRCDFRTTYKAKEKGVKLPG
AHFVDHCIEILSHDKDYNKVKLYEHAVAHSGLPDNARRGGGGSGGGGSTGPSLIPPVTFEVKA
ESLGIPQKMQLKVDVESGKLIIKKSKDGSEDKFYSHKKILQLIKSQKFLNKLVILVETEKEKILRKE
YVFADSKKREGFCQLLQQMKNKHSEQPEPDMITIFIGTWNMGNAPPPKKITSWFLSKGQGKTR
DDSADYIPHDIYVIGTQEDPLSEKEWLEILKHSLQEITSVTFKTVAIHTLWNIRIVVLAKPEHENRIS
HICTDNVKTGIANTLGNKGAVGVSFMFNGTSLGFVNSHLTSGSEKKLRRNQNYMNILRFLALGD
KKLSPFNITHRFTHLFWFGDLNYRVDLPTWEAETIIQKIKQQQYADLLSHDQLLTERREQKVFLH
FEEEEITFAPTYRFERLTRDKYAYTKQKATGMKYNLPSWCDRVLWKSYPLVHVVCQSYGSTSD
IMTSDHSPVFATFEAGVTSQFVSKNGPGTVDSQGQIEFLRCYATLKTKSQTKFYLEFHSSCLES
FVKSQEGENEEGSEGELVVKFGETLPKLKPIISDPEYLLDQHILISIKSSDSDESYGEGCIALRLE
ATETQLPIYTPLTHHGELTGHFQGEIKLQTSQGKTREKLYDFVKTERDESSGPK*

Human SHIP1 (INPP5D) 1-1188aa domain organization:

SH2 Domain: 5-101aa
PH-R Domain: 292-401aa
Phosphatase Domain: 401-866aa

PPtase-C2 linker
C2 Domain: 725-863aa
C- terminus/proline-rich: 920-1148aa

MVPCWNHGNITRSKAEELLSRTGKDGSFLVRASESISRAYALCVLYRNCVYTYRILPNEDDKFT
VQASEGVSMRFFTKLDQLIEFYKKENMGLVTHLQYPVPLEEEDTGDDPEEDTESVVSPPELPP
RNIPLTASSCEAKEVPFSNENPRATETSRPSLSETLFQRLQSMDTSGLPEEHLKAIQDYLSTQL
AQDSEFVKTGSSSLPHLKKLTTLLCKELYGEVIRTLPSLESLQRLFDQQLSPGLRPRPQVPGEA
NPINMVSKLSQLTSLLSSIEDKVKALLHEGPESPHRPSLIPPVTFEVKAESLGIPQKMQLKVDVE
SGKLIIKKSKDGSEDKFYSHKKILQLIKSQKFLNKLVILVETEKEKILRKEYVFADSKKREGFCQLL
QQMKNKHSEQPEPDMITIFIGTWNMGNAPPPKKITSWFLSKGQGKTRDDSADYIPHDIYVIGTQ
EDPLSEKEWLEILKHSLQEITSVTFKTVAIHTLWNIRIVVLAKPEHENRISHICTDNVKTGIANTLG
NKGAVGVSFMFNGTSLGFVNSHLTSGSEKKLRRNQNYMNILRFLALGDKKLSPFNITHRFTHLF
WFGDLNYRVDLPTWEAETIIQKIKQQQYADLLSHDQLLTERREQKVFLHFEEEEITFAPTYRFER
LTRDKYAYTKQKATGMKYNLPSWCDRVLWKSYPLVHVVCQSYGSTSDIMTSDHSPVFATFEA
GVTSQFVSKNGPGTVDSQGQIEFLRCYATLKTKSQTKFYLEFHSSCLESFVKSQEGENEEGSE
GELVVKFGETLPKLKPIISDPEYLLDQHILISIKSSDSDESYGEGCIALRLEATETQLPIYTPLTHHG
ELTGHFQGEIKLQTSQGKTREKLYDFVKTERDESSGPKTLKSLTSHDPMKQWEVTSRAPPCSG
SSITEIINPNYMGVGPFGPPMPLHVKQTLSPDQQPTAWSYDQPPKDSPLGPCRGESPPTPPGQ
PPISPKKFLPSTANRGLPPRTQESRPSDLGKNAGDTLPQEDLPLTKPEMFENPLYGSLSSFPKP
APRKDQESPKMPRKEPPPCPEPGILSPSIVLTKAQEADRGEGPGKQVPAPRLRSFTCSSSAEG
RAAGGDKSQGKPKTPVSSQAPVPAKRPIKPSRSEINQQTPPTPTPRPPLPVKSPAVLHLQHSK
GRDYRDNTELPHHGKHRPEEGPPGPLGRTAMQ*
